# Root Secreted Metabolites and Proteins Are Involved in the Early Events of Plant-Plant Recognition Prior to Competition

**DOI:** 10.1371/journal.pone.0046640

**Published:** 2012-10-02

**Authors:** Dayakar V. Badri, Clelia De-la-Peña, Zhentian Lei, Daniel K. Manter, Jacqueline M. Chaparro, Rejane L. Guimarães, Lloyd W. Sumner, Jorge M. Vivanco

**Affiliations:** 1 Department of Horticulture and Landscape Architecture and Center for Rhizosphere Biology, Colorado State University, Fort Collins, Colorado, United States of America; 2 U.S. Department of Agriculture - Agricultural Research Service, Soil-Plant-Nutrient Research Unit, Fort Collins, Colorado, United States of America; 3 Bio-Rad Laboratories, Hercules, California, United States of America; 4 The Samuel Roberts Noble Foundation, Plant Biology Division, Oklahoma, United States of America; Uni. of South Florida, United States of America

## Abstract

The mechanism whereby organisms interact and differentiate between others has been at the forefront of scientific inquiry, particularly in humans and certain animals. It is widely accepted that plants also interact, but the degree of this interaction has been constricted to competition for space, nutrients, water and light. Here, we analyzed the root secreted metabolites and proteins involved in early plant neighbor recognition by using *Arabidopsis thaliana* Col-0 ecotype (Col) as our focal plant co-cultured *in vitro* with different neighbors [*A. thaliana* Ler ecotype (Ler) or *Capsella rubella* (Cap)]. Principal component and cluster analyses revealed that both root secreted secondary metabolites and proteins clustered separately between the plants grown individually (Col-0, Ler and Cap grown alone) and the plants co-cultured with two homozygous individuals (Col-Col, Ler-Ler and Cap-Cap) or with different individuals (Col-Ler and Col-Cap). In particularly, we observed that a greater number of defense- and stress- related proteins were secreted when our control plant, Col, was grown alone as compared to when it was co-cultured with another homozygous individual (Col-Col) or with a different individual (Col-Ler and Col-Cap). However, the total amount of defense proteins in the exudates of the co-cultures was higher than in the plant alone. The opposite pattern of expression was identified for stress-related proteins. These data suggest that plants can sense and respond to the presence of different plant neighbors and that the level of relatedness is perceived upon initial interaction. Furthermore, the role of secondary metabolites and defense- and stress-related proteins widely involved in plant-microbe associations and abiotic responses warrants reassessment for plant-plant interactions.

## Introduction

How organisms recognize each other has been at the forefront of inquiry ever since people began to wonder when babies begin to perceive themselves as unique individuals separate from their mothers. In the 70 s, studies were conducted in which chimpanzees were found to recognize themselves in mirror reflections but monkeys failed to do likewise [1]. These reports led to a flurry of experiments that showed similar cognitive events in animals such as elephants, pigeons, and dolphins among others; and to detailed studies involving comparative brain scans between humans and chimpanzees [2]. Ultimately, these studies and others had an effect on the development of human consciousness as a scientific topic. However, the molecular and biochemical mechanisms related to the ability of organisms to differentiate self vs. non-self has not progressed as rapidly due to the lack of appropriate models devoid of all layers of complexity.

The ability of organisms to differentiate each other has been studied at various levels in the context of self- and non-self recognition. One study found a molecular determinant, the ids (identification of self) locus, on the bacterium *Proteus mirabilis* that allows colonies from the bacteria to differentiate between self and non-self by the development of boundaries between the colonies [3]. However, the mechanism of action of this locus was not reported. In plants, Mahall and Callaway [4] first reported self and non-self recognition based on counting the number of roots of the desert shrub *Ambrosia dumosa* when it encounters a clonal individual as compared to a different individual from the same species. Subsequent studies demonstrated self/non-self recognition of roots in different plant species, such as strawberry [5], peas [6] and buffalo grass [7]. All these studies were conducted at an ecological level and root allocation differences between plants grown with strangers compared to siblings were considered measurements of recognition [8]. The mechanism behind self/non-self root discrimination still remains obscure but it has been suggested that root exudates play a role in this recognition [9], [10].

Most biochemical and molecular studies related to plant recognition have focused on the aerial parts of plants, describing how volatiles from leaves and flowers can establish communication with other plants [11], [12]. However, the plant-plant communication in these interactions is induced only when neighboring plants release volatile organic compounds (VOCs), such as nonanal, (Z)-3-hexanol, (E)-2-hexanal, (Z)-3-hexanyl acetate and methyl jasmonate, upon attack by insects or pathogens [11], [13]–[15]. Roots comprise a significant part of a plant’s structure; however, little has been done to understand root recognition of plant neighbors at the biochemical and molecular level. Previous reports have described that roots secrete small molecules, volatiles, and proteins that act as signaling or recognition molecules in plant-microbe interactions [16], [17] [18], [19]. In addition, it was reported that the proteins in the root exudates are differentially secreted depending upon the presence of compatible or incompatible bacteria and vice versa the proteins released by specific bacterium depend on the identity of the plants (and roots) that they interact with [16].

It was reported that in ants [20] and *Drosophila melanogaster* [21] individuals gene expression at the whole genome level was affected by the genotypic composition of other group members. However, these studies did not investigate whether within-group relatedness affected patterns of gene expression. Recently, two reports determined how patterns of gene expression in the plant roots are affected by the kin structure of the group [22], [23]. Both studies used different experimental designs and found contrasting results. Masclaux et al., [22] did not find significant differential gene expression in the roots of the focal plant, which was surrounded by their kin or non-kin neighbors after 12 days of co-culture; but found significant differences in their biomass as a function of the interacting genotype. In contrast, Biedrzycki and Bais [23] found significant gene expression differences in ATP/GST transporter, auxin/auxin related, secondary metabolite, and pathogen response genes when the focal plant was exposed to root secretions of its own, kin, or a stranger. Another recent report demonstrated that plants could communicate by unknown mechanisms other than those mediated by light and chemicals [24].

Previous studies have shown that plants exhibit competition behavior towards other plants by producing more number of roots. When a given plant encounters an individual from a different species the degree of competition (by means of measuring root production) increases compared to when the plant encounters an individual from the same species [4]–[7]. Based on this information, the present report was designed to obtain a biochemical understanding of the means by which roots of *A. thaliana* respond to different plant neighbors. We choose to look at root exudates to determine whether neighbor identity can influence the quantity and/or quality of signals secreted by the roots. For this purpose, we selected *A. thaliana* ecotypes Col-0 and Landsberg erecta (Ler), and *Capsella rubella* (Cap) due to their similarities/differences in their genome composition. *A. thalaiana* ecotypes Col and Ler have close genome relationship, but are not identical, based on genetic diversity and genome sequence comparison analyses [25], [26]. Similarly, Cap belongs to the same family (Brassicae) as Arabidopsis and has >33% similarity at the nucleotide sequence level with *A. thaliana* [27], [28]. With this combined information, we hypothesized that the competitive ability of the *A. thaliana* ecotype Col-0 would be higher when grown with Cap than when grown with ecotype Ler or with the same individual (Col-0). For this purpose, *A. thaliana* Col-0 ecotype was co-cultured with different neighbors exhibiting different degrees of relatedness and assumed levels of competition. *A. thaliana* Col-0 ecotype was cultured with a similar homozygous Col-0 individual, with Ler ecotype or with Cap. The interaction time between the plant individuals was kept to a minimum (co-cultured three days) to identify early biochemical markers in the root exudates that are involved in neighbor differentiation.

## Materials and Methods

### Plant Material and Growth Conditions

*Arabidopsis thaliana* Col-0 ecotype (Col), *A. thaliana* Lansberg erecta ecotype (Ler) and *Capsella rubella* (Cap) seeds were purchased from Lehle Seeds (Round Rock, TX) and ABRC (Ohio State University, USA). The seeds were surface sterilized with 3% (v/v) sodium hypochlorite for two minutes followed by three washes with sterile distilled water. The seeds were germinated on solidified MS [29] media supplemented with 3% (w/v) sucrose in a growth chamber at 25±2°C and 16/8 h day/night photoperiod. Each seven-day-old individual plant was transferred to a six well plate containing 5 ml of liquid MS media supplemented with 1% (w/v) sucrose and placed on a shaker set at 70 rpm, 24±2°C under photoperiod of 16/8 h. After two weeks each plant was gently washed with sterile distilled water and transferred into a Magenta box containing 10 ml of liquid MS media supplemented with 1% (w/v) sucrose. As a control, one plant was transferred into a Magenta box containing 10 ml of liquid MS media supplemented with 1% sucrose. The sterility of the MS media was checked by visual observation. For the recognition studies, two homozygous plants or two different plants with varying degrees of relatedness were transferred into a Magenta box containing 20 ml of liquid MS media supplemented with 1% (w/v) sucrose. The root exudates in these conditions were collected after three days of continuous secretion and then subjected to proteomic analyses. It should be noted that these exudates represent the combined protein secretions from both plants of the co-culture. For the controls, the root exudates secreted from 40 plants were pooled together as one replicate and for the neighbor interactions the root exudates secreted from 40 co-cultures were pooled together as one replicate. The whole experiment was performed with three biological replicates for each treatment. In the co-cultures, both individuals were placed together in the Majenta box which allowed the individuals to experience physical contact through their roots.

### Temporal Collection of Root Exudates

Plantlets were grown in Magenta boxes as described above. Three days post transfer, the exudates of 40 plants or 40 co-cultures were collected, pooled and centrifuged at 8,000 g for 15 min at 4°C to remove the root sheathing. The supernatants of Col, Col-Col, Col-Ler, Col-Cap, Ler, Ler-Ler, Cap and Cap-Cap were filtered through a 0.2 µm syringe filter and the filtrate was concentrated to 20 ml by lyophilization. The concentrated root exudates were desalted and further concentrated to 500 µl by passing through Amicon Ultra Centrifugal Filter Devices (MWCO: 5000 Da, Millipore) and used for proteomic studies. Root-exuded proteins were stored at −80°C until needed. For each combination of plants, three biological replicates (n = 3; 120 plants in total) were used. The protein concentration of the samples were determined as described by Bradford [30] using a protein assay kit (Bio-Rad Laboratories) and bovine serum albumin (BSA) as a standard.

### Metabolomics

#### Extraction of phytochemicals

After separating the proteins from the root exudates by using Amicon ultra centrifugal devices, the fraction containing phytochemicals was freeze dried (Labconco Kansas City, MO, USA), dissolved in 10 ml of distilled water and the pH was adjusted to 3.0 with 1N HCl. The liquid was partitioned three times with an equal volume of ethyl acetate (EtOAc, Fisher Scientific). All three EtOAc fractions were pooled and the remaining water residues were removed using sodium sulfate as a drying agent. The dried concentrate was dissolved in 100 µl of methanol (MeOH) for subsequent HPLC-MS analysis.

#### High-Performance Liquid Chromatography (HPLC) and mass spectrometry

The phytochemicals extracted from liquid media were chromatographed by gradient elution on a 150 mm×4.6 mm reverse phase, C18 column (Dionex). The chromatographic system (Dionex Co., Sunnyvale, CA) consisted of two P680 pumps connected to an AS1–100 automated sample injector and detected at 280 nm with a UV-visible detector. Mass determination of the peaks was analyzed by an MSQ-MS detector system (Thermo Electron Co., Waltham, MA). A gradient was applied for all separations with a flow rate of 0.7 ml min-1. The gradient was as follows: 0–3 min, 90.0% water and 10% methanol; 3–43 min, 10.0 to 90% (v/v) methanol, 90 to 10% (v/v) water; 43–51 min, 90.0% (v/v) methanol and 10% (v/v) water.

#### HPLC-MS chromatograms analyses

Alignment of the chromatographic datasets by retention time and mass was performed using the XCMS software (https://xcmsonline.scripps.edu/) [31] to generate an aligned data matrix suitable for statistical analyses. Analyte features were labeled by their retention time and mass, and exported to Metaboanalyst a web server tool [32] for multivariate analysis. Data filtering was performed by interquantile range method followed by quantile normalization within replicates after log transformation. Principal Components Analysis (PCA) and significant features identification was performed for all treatments together. Cluster analysis was performed by using the Ward method. Pattern finding was performed based on Pearson correlation values.

### Proteomics

#### Two-dimensional sodium dodecyl sulfate polyacrylamide gel electrophoresis (2-DE) separations

2-DE was used to separate [33] and quantify the proteins secreted in the root exudates following the different treatments. Four hundred and fifty micrograms of total exuded proteins from each biological triplicate of the treatments Col, Col-Col, Col-Ler, Col-Cap, Ler, Ler-Ler, Cap and Cap-Cap were analyzed independently by 2-DE following the protocol described by De-la-Peña et al., [16]. Briefly, the exuded proteins were precipitated using 12.5% (w/v) TCA plus 1% 2-mercaptoethanol and incubated at −20°C for 45 min. Immobilize pH gradient strips (IPG: ImmobilineTM Dry Strips, 24 cm, pH 3–10 non-linear, Amersham Biosciences) were rehydrated for 12 h at 20°C with 450 µg of protein in 500 µl of 2-DE solubilization buffer consisting of 7 M urea, 2 M thiourea, 3% (w/v) CHAPS, 2% (v/v) Triton X-100, 20 mM DTT and 0.5% ampholytes. Isoelectric focusing (IEF) of proteins was performed using the following step gradient: 500 volts for one hour, 1000 volts for one hour, and 8000 volts until a total of 50,000 V-hr had been achieved. The IPG strips were then loaded onto SDS polyacrylamide gel (12.5% T, 1 mm thick) and second dimensional electrophoresis separation performed at 110 mA overnight at 10°C. Separated proteins were visualized using silver staining [34].

#### Quantitative image and protein analyses

For spot detection, quantification, background subtraction and comparative analysis between the different ecotype interactions, two bioinformatics approaches were used. The gels were digitally imaged with a FluorS (Bio-Rad Laboratories) equipped with a 12-bit camera by using first the Phoretix 2D Expression software (v 2005, Nonlinear Dymanics, Durham, NC) from three different biological replicate gels for each treatment. A spot volume was calculated and each spot was assigned a normalized spot volume as a relative portion of the total value prior to analysis by ANOVA. Each individual protein spot was then matched with the identical protein spot from each replicate gel. For the 2DE gels analysis, we also used the software PDQuest Advanced (Bio-Rad Laboratories) version 8.0.1. Three biological replicate gels of each treatment were normalized using local regression model Loess. Quantitative analysis set for each sample type was created using triplicate gels. Proteins that consistently displayed 3-fold or greater increase or decreased expression when compared to the control (Col) were validated and reported. Proteins that remained unchanged, up to 1.5-fold were also validated and reported. Boolean analysis set was created to evaluate intersections of two or more sets of spots. Cumulative spot intensities were calculated for each of the functional categories (e.g., defense, secretory, peroxidase, etc.) found in the protein profiles (128 identified proteins). Interaction-specific differences between protein profiles were tested using a multivariate ANOVA (SAS Vers. 9.2, Cary, NC, USA) and the exudate profiles comprised all 128 identified proteins. Alternatively, a separate ANOVA was used to test interaction-specific differences for the cumulative spot intensities of each categorized proteins.

#### In-gel trypsin digestion and nano LC-QTOF/MS/MS analyses

Differentially accumulated 2-DE protein spots observed during the interactions between plants of the same individual and plants of different individuals were excised and separately digested with trypsin prior to mass spectrometry analyses as part of the protein identification process. Silver-visualized protein spots were manually excised from the gels. These gel plugs were transferred to polypropylene 96-well plates and destained according to Sumner et al., [35]. The gel spots were dehydrated with 25 µl of acetonitrile (ACN) for 15 min at room temperature and ACN removed. The gel plugs were dried under vacuum and rehydrated in 20 µl of sequencing-grade modified bovine trypsin (10 ng/µl in 25 mM ammonium biocarbonate, Roche Diagnostics). After rehydration for 30 min on ice, excess trypsin solution was removed, and 15 µl of 25 mM ammonium bicarbonate was added to each well to prevent dehydration during incubation. Proteolysis was allowed to continue for 13 hrs at 37°C and stopped by adding 15 µl of 10% formic acid. All peptide extract fractions were pooled, concentrated until dry and resuspended in a 50∶50 (%v/v) water-acetonitrile solution containing a final concentration of 0.1% formic acid.

The protein digests were analyzed using a nanoscale HPLC system (LC Packings, San Francisco, CA) consisting of an autosampler (Famos), a precolumn switching device (Switchos), and a nano HPLC pump system (Ultimate). Samples (5 µl) were loaded onto a C18 precolumn (0.3-mm inner diameter×1.0 mm, 100 Å, PepMap C18, LC Packings) for desalting and concentrating at a flow rate of 50 µl/min using mobile phase A (5% ACN and 95% water containing 0.1% formic acid). The desalted peptides were then eluted from the precolumn and separated on a nano analytical C18 column (75- µm ID X 15 cm, 100 Å, PepMap C18, LC Packings) at a flow rate of 200 nL/min. Peptides were eluted with a linear gradient of 5–40% mobile phase B (95% ACN and 5% water containing 0.08% formic acid) over 40 min. The separated peptides were directly analyzed with an ABI QSTAR Pulsar I hybrid quadrupole time-of-flight mass spectrometer (QTOF-MS; Applied BioSystems) equipped with a nanoelectrospray ionization source (Protana). QTOF-MS and tandem mass spectral data were acquired using information-dependent acquisition (IDA) with the following settings: charge state selection from 2 to 5, an intensity threshold of 10 counts/s for tandem experiments, and a collision energy setting automatically determined by the IDA based on the m/z values of each precursor ion.

#### Database queries and protein identification

For protein identification, the acquired mass spectral data were queried against the NCBI non-redundant protein database (NCBInr), downloaded on 4-22-2003, using the MASCOT (version 2.2, Matrix Science Ltd., London, UK) search engine [36], [37] with the following settings, a mass tolerance of 100 ppm, one trypsin missed cleavage allowance, and two variable amino acid modifications, i.e., methionine oxidation and cysteine carbamidomethylation. Only protein identifications with a molecular weight search (MOWSE) score greater than the generally accepted significant threshold (determined at 95% confidence level as calculated by MASCOT; p<0.05) and at least two matched peptides are reported in this study.

## Results

### Root Exuded Metabolites

**Figure 1:**
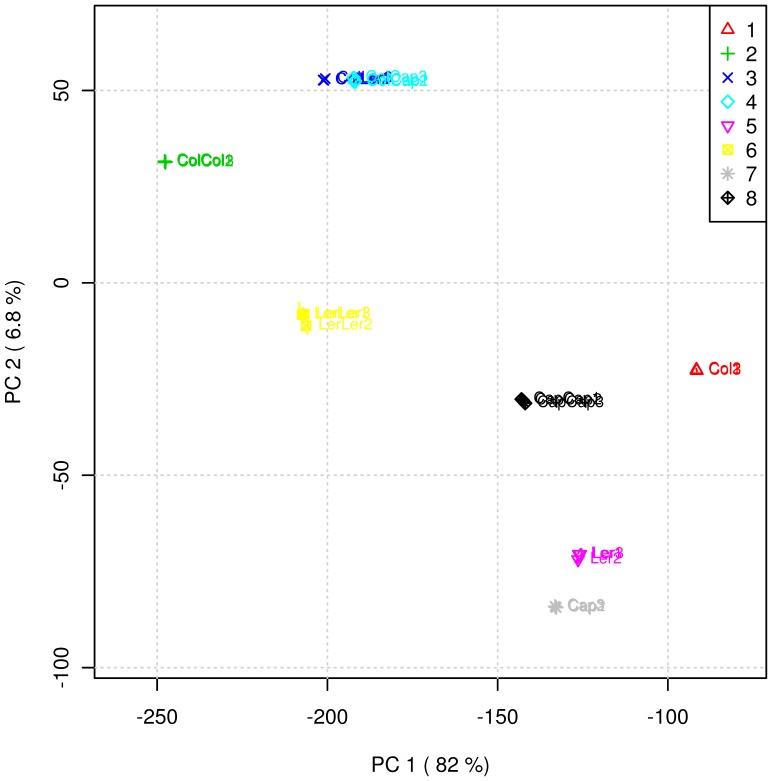
Principal Component Analyses (PCA) of the root secreted secondary metabolites of the plants grown alone, co-cultured with homozygous individuals and co-cultured with different plants. 1: Col; 2: Col-Col; 3: Col-Ler, 4: Col-Cap, 5: Ler, 6: Ler-Ler, 7: Cap, 8: Cap-Cap.

**Figure 2:**
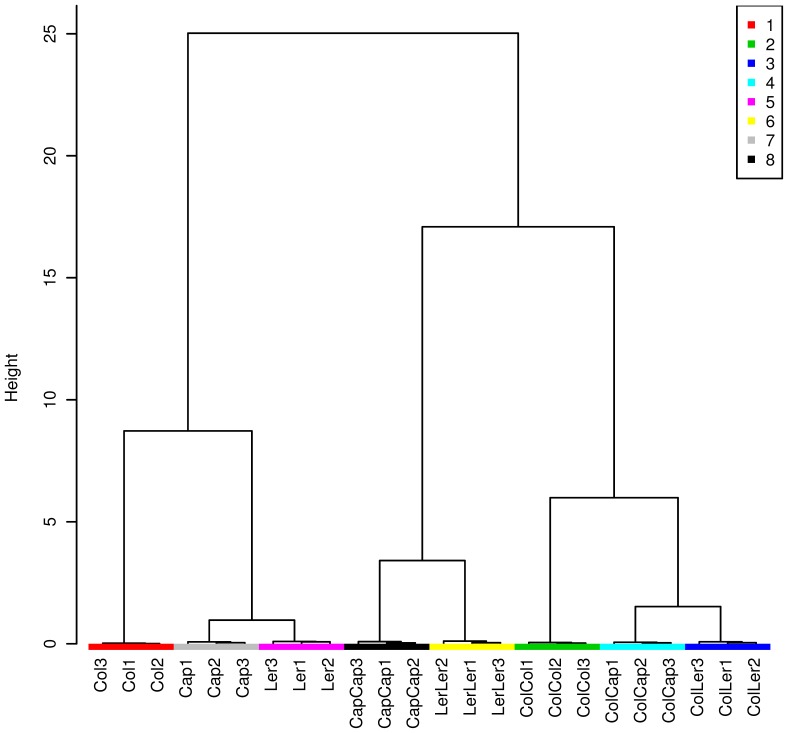
Cluster analyses of the root secreted secondary metabolites of the plants grown alone, co-cultured with homozygous individuals and co-cultured with different plants by the Ward method. 1: Col; 2: Col-Col; 3: Col-Ler, 4: Col-Cap, 5: Ler, 6: Ler-Ler, 7: Cap, 8: Cap-Cap.

We first analyzed the secondary metabolites present in the root exudates of the different treatments. Principal component analysis (PCA) and cluster analysis by the Ward method were performed on the qualitative and quantitative data of 191 mass features. Based on the PCA analyses, we observed clear groupings between the plants grown individually (Col-0, Ler and Cap grown alone) and the plants co-cultured with two homozygous individuals (Col-Col, Ler-Ler and Cap-Cap) or with different individuals (Col-Ler and Col-Cap) (Figures 1 & 2). Further, we identified specific patterns based on the Pearson correlation values: the mass features (545.21 at retention time (RT) 36.16, 746.68 at RT 48.03, 301.20 at RT 49.4, 598.55 at RT 49.97, 745.66 at RT 50.75 and 721.62 at RT 52.68) were specifically found only in the co-cultures of Col-Col, Col-Ler and Col-Cap (Table S1) but not in other co-cultures or plants grown alone. Similarly, the mass features (167.17 at RT 4.08, 120.23 at RT 28.8, 899.53 at RT 29.82, 621.5 at RT 46.64, 949.64 at RT 29.08 and 904.55 at RT 29.26) were only observed in the co-cultures of Col-Col, Ler-Ler and Cap-Cap but not in other co-cultures or plants grown alone. These data clearly suggests that plants secrete specific metabolites depending upon the identity of the plant neighbor.

### Overall Quantification of Root Secretion of Proteins

We also analyzed the proteins secreted in the root exudates of the different treatments were detected using 2-DE-gel electrophoresis (Figure S1) and identified by mass spectrometry (Table S2). The total number of protein spots observed in the 2-DE-gels included a total of 426 (Col), 343 (Ler) and 123 (Cap) protein spots when plants were grown individually and 536 (Col-Col), 265 (Ler-Ler) and 190 (Cap-Cap) upon co-culturing of two homozygous individuals. Similarly, 445 (Col-Ler) and 368 (Col-Cap) protein spots were identified when co-culturing two different individuals (Figure S2). The majority of these proteins were found in two or more root exudate profiles (Figure S2). For instance, Col commonly shared 115 and 43 proteins spots with Ler and Cap respectively, when plants were grown individually. Similarly, when co-cultured with two different individuals, Col-Col shared 195 and 175 protein spots with Col-Ler and Col-Cap, respectively.

**Figure 3:**
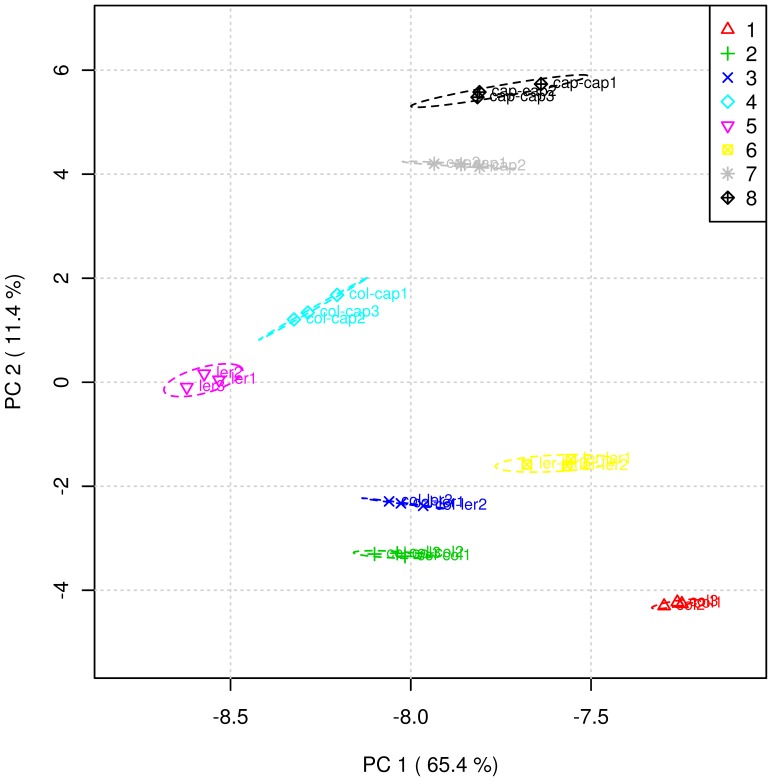
Principal Component Analyses (PCA) of the 128 root secreted proteins of the plants grown alone, co-cultured with homozygous individuals and co-cultured with different plants. 1: Col; 2: Col-Col; 3: Col-Ler, 4: Col-Cap, 5: Ler, 6: Ler-Ler, 7: Cap, 8: Cap-Cap.

**Figure 4:**
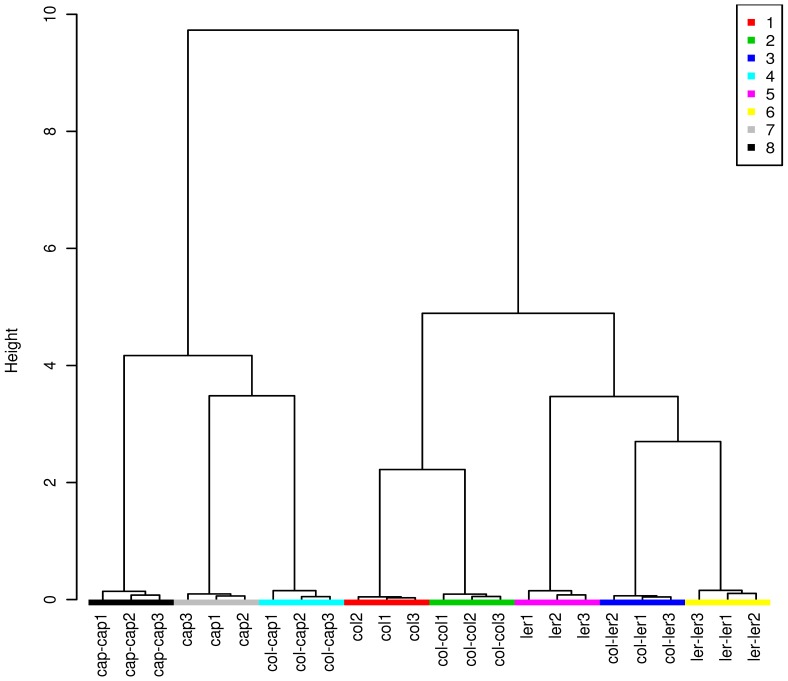
Cluster analyses of the 128 root secreted proteins of the plants grown alone, co-cultured with homozygous individuals and co-cultured with different plants by the Ward method. 1: Col; 2: Col-Col; 3: Col-Ler, 4: Col-Cap, 5: Ler, 6: Ler-Ler, 7: Cap, 8: Cap-Cap.

A total of 128 protein spots were excised for identification based on the following criteria: 1) protein spots observed in all three replicates of each treatment, 2) protein spots that showed changes both quantitatively and qualitatively between the treatments, and 3) a spot was present at a sufficient concentration for nano LC-QTOFMS identification. PCA and cluster analysis by the Ward method were performed on the qualitative and quantitative data of the 128 proteins with the help of the web server tool Metaboanalyst [32]. We observed clear groupings between plants grown individually, co-cultured with the homozygous individuals, and with the different individuals (Figures 3 & 4 and Table S2). For example, Col, Ler and Cap plants grown alone are clustered together with the plants co-cultured with their homozygous individuals (Col-Col, Ler-Ler and Cap-Cap) (Figure 4). Arabidopsis Col co-cultured with different individuals (Col-Ler and Col-Cap) clustered distinctively indicating that for the most part proteins are specifically secreted based on the identity of the plant neighbor. Similarly, MANOVA analysis showed that the protein profiles differed significantly (p-value <0.0001) between treatments (Tables S3–S6).

### Root Secretion by Individual Plants

**Figure 5:**
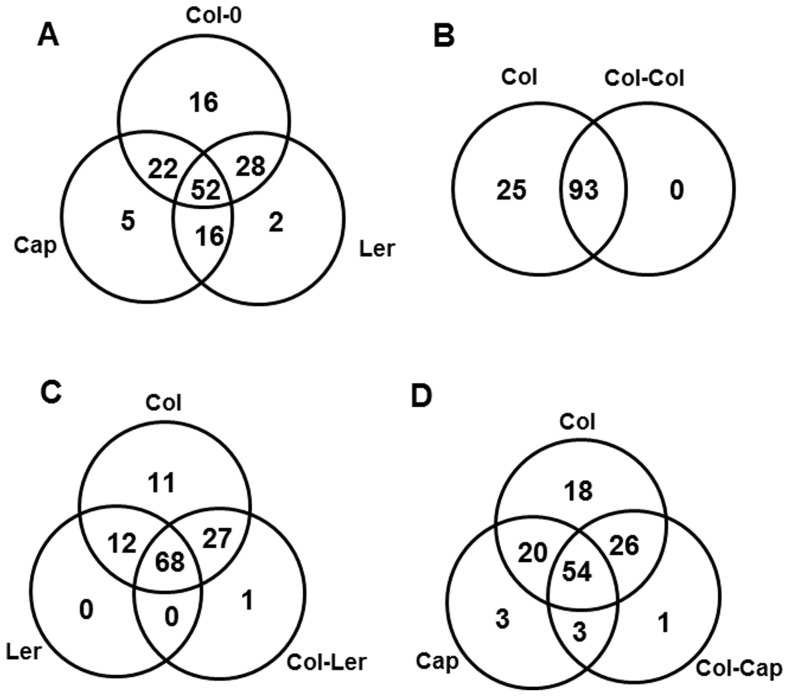
Venn diagram showing the qualitative differences between the root secreted proteins of the plants grown alone and the different co-culture treatments. (A) Comparison between the plants Col, Ler and Cap grown alone. (B) Comparison between the plant Col grown alone and co-cultured with another Col. (C) Comparison between the plants Col and Ler grown alone with the co-culture Col-Ler. (D) Comparison between the plants Col and Cap grown alone with the co-culture Col-Cap. The numbers present inside the circles are the number of specific proteins present in the particular treatment but not in other treatments. The numbers present in the intersection are the number of proteins shared between the treatments.

Qualitative differences in the protein secretion profiles were observed when Col, Ler and Cap were grown individually (Figure 5A). For example, 16 proteins were only secreted by Col, which included peroxidases (spots # 19, 20, 21, 49, 51 & 53), myrosinase-binding proteins (spots # 2, 32, 98), defense-related proteins (spots # 38, 57, 76), and other proteins like CoR13 (spot # 26), mitochondrial NAD-dependent malate dehydrogenase (spot # 71) and unknown proteins (spots # 95 & 124). Similarly, two myrosinase-binding proteins (spots # 125 and 127) and five proteins [peroxidases (spots # 115 &116), defense-related proteins (spots # 120 & 122), and one unknown protein (spot # 117)] were secreted by Ler ecotype and Cap, but not Col. In contrast, a greater number of proteins were common to two or more of the plants examined. For example, 52 proteins were common to all 3 ecotypes, 28 common to Col and Ler and 22 common to Col and Cap (Figure 5A).

### Proteins Secreted Differentially upon Co-culturing of Two Homozygous Individuals

**Figure 6:**
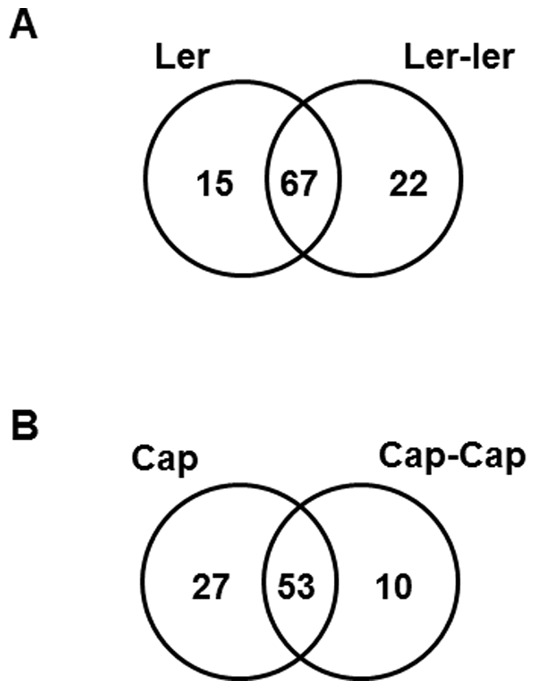
Venn diagram showing the qualitative differences between the root secreted proteins of the co-cultures of the homozygous individuals of Ler and Cap. (A) Comparison between Ler and Ler-Ler. (B) Comparison between Cap and Cap-Cap. The numbers of proteins unique to Ler are represented inside the circles and the numbers of proteins shared between the treatments are represented in the intersections.

Twenty-five proteins were secreted by Col when grown alone, but were absent when co-cultured with the same homozygous individual. These proteins were: glycosyl hydrolases (spots # 14 & 16), peroxidases (spots # 17, 19, 49,96, 100 & 102), defense-related proteins (spots # 46, 54, 57, 83 & 84), secretory protein-related (spot # 74), myrosinase binding protein-related (spots # 92 & 97), xylosidase (spot # 128) and proteins (spots # 22, 23, 43, 61, 76, 79, 95 & 111) of unknown function (Figure 5B). Similar trends were also observed with Ler co-cultured with Ler and Cap co-cultured with Cap. For instance, 15 proteins [defense-related proteins (spots # 4, 30, 54, 80, & 88), peroxidases (spots # 96 & 100), myrosinase binding protein-related (spots # 92 & 108), secretory protein (spot # 74 & 94), metallendopeptidase (spot # 6), XYL4 (spot # 8), XTR6 (spot # 70) and protein (spot # 81) of an unknown function] were absent when Ler co-cultured with another Ler (Figure 6A). Similarly, 27 proteins [defense-related proteins (spots # 4, 46, 54, 62, & 86), peroxidases (spots # 9, 47, 60, 110 & 116), myrosinase binding protein-related (spots # 27, 42, 92, & 105), hydrolases (spots # 13, & 39), protein kinase (spot # 25), XTR6 (spot # 70), MER15B (spot # 78), Meri-5 (spot # 79), secretory protein (spot # 94) and proteins (spots # 18, 33, 50, 61, 87 & 109) of unknown function] were absent when Cap was co-cultured with another Cap (Figure 6B).

Unlike the Col-Col treatment, new proteins were observed in the co-culture treatments of Ler-Ler compared with Ler grown alone (Figure 6A) and Cap-Cap compared with Cap grown alone (Figure 6B). For instance, 22 new proteins [myrosinase binding protein-related (spots # 2, 98 & 107), hydrolases (spots # 16 & 82), peroxidases (spots # 49, 51, 53, & 60), defense-related proteins (spots # 38, 62 & 84), protein kinase (spot # 25), NAD dependent malate dehydrogenase (spot # 71), XYL6 (spot # 73), Meri-5 (spot # 79) and proteins (spots # 18, 22, 43, 93, 109 & 124) of unknown function] were present in the co-culture treatment of Ler-Ler compared with Ler grown alone. Similarly, 10 new proteins [defense-related proteins (spots # 5, 45, 80 & 83), peroxidases (spots # 56 & 100), secretory protein (spot # 74), XTR6 (spot # 71), ATGLX1 (spot # 76) and a protein (spot # 89) of unknown function] were present in the co-culture treatment of Cap-Cap compared with Cap grown alone (Table S1).

### Proteins Secreted Differentially upon Co-culturing with Dissimilar Individuals

Qualitative differences in protein secretions were observed when Col was co-cultured with different individuals (Col-Ler and Col-Cap) and compared with their partners (Col, Ler and Cap) grown alone. For instance, Col secretes 11 unique proteins [peroxidases (spots # 19, 20, 21, 32 & 60), hydrolases (spot # 16), myrosinase binding protein-related (spot # 27), ATGLX1 (spot # 76), Meri-5 (spot # 79), SJCHGCO8196 (spot # 95) and a protein (spot # 43) of unknown function] present only in Col grown alone and not in Ler or in the co-culture of Col-Ler (Figure 5C). Besides these, there are 27 proteins [defense-related proteins (spots # 38, 57, 62, & 84), myrosinase binding protein related (spots # 2, 98 & 107), hydrolases (spots # 3, 29 & 82), peroxidases (spots # 47, 49, 51, 53 & 106), protein kinase (spot # 25), COR13 (spot # 26), NAD dependent malate dehydrogenase (spot # 71), XYL6 (spot # 73) and proteins (spots # 18, 22, 33, 50, 93, 109, 121 & 124) of unknown function] that were commonly shared between Col grown alone and the co-culture of Col-Ler but absent in Ler grown alone. Among these 27 proteins, nine proteins [defense-related proteins (spots # 57, 62, 65 & 84), myrosinase binding protein related (spot # 107), NAD dependent malate dehydrogenase (spot # 71), XYL6 (spot # 73) and proteins (spots # 109 & 121) of unknown function] were secreted at higher levels in the co-culture of Col-Ler compared to Col grown alone (Table S2). It is worth mentioning that these proteins are completely absent in Ler grown alone and this result suggests that these proteins originate from Col and are secreted at higher levels in response to the neighbor. In addition, all nine of these proteins were secreted at low levels or absent in the co-culture of Col-Col compared with Col-Ler. In particular, defense-related proteins (spots # 57 & 84) are completely absent in the co-culture of Col-Col, but present at higher levels in the co-culture of Col-Ler compared with Col grown alone.

Similar trends were also observed when comparing Col, Cap and the co-culture of Col-Cap. For instance, Col secreted 18 unique proteins [peroxidases (spots # 17, 32, 49, 55, 96, 100 & 106), hydrolases (spot # 14), myrosinase binding protein-related (spots # 97, 98 & 108), defense-related proteins (spots # 38, 80 & 83), ATGLX1 (spot # 76), SJCHGCO8196 (spot # 95), LOC683313 protein (spot # 111) and a protein (spot # 114) of unknown function] were present only in Col grown alone and not in Cap or in the co-culture of Col-Cap (Figure 5D). Besides these, there are 26 proteins [defense-related proteins (spots # 5, 45 & 57), myrosinase binding protein related (spots # 1, 2, 99 & 101), hydrolases (spot # 11), peroxidases (spots # 19, 20, 21, 37, 44, 48, 51, 53, 56, & 103), secretory protein (spot # 74), XYL4 (spot # 8), pepsin A (spot # 15), COR13 (spot # 26), NAD dependent malate dehydrogenase (spot # 71), XYL4 (spot # 128) and proteins (spots # 89 & 124) of unknown function] were commonly shared between Col grown alone and co-culture of Col-Cap but absent in Cap grown alone. Among these 26 proteins, nine proteins [defense-related proteins (spot # 45), myrosinase binding protein related (spots # 99 & 101), peroxidases (spots # 19, 20 & 103), secretory protein (spot # 74), XYL4 (spot # 128) and protein (spot # 124) of unknown function] were secreted at higher levels in the co-culture of Col-Cap compared to Col grown alone (Table S2). These nine proteins are completely absent in Cap grown alone, suggesting that these proteins originate from Col and were secreted at higher levels in response to the neighbor. Among those nine proteins, three proteins (spots # 19, 74 & 128) were secreted at low levels or absent in the co-culture of Col-Col compared with Col-Cap.

In addition, we found that specific proteins were secreted at higher levels depending upon their neighbor identity. For example, the secretion levels of subtilisin-like proteases (spots # 4 & 5) are higher in the co-culture Col-Col compared with other co-culture treatments and the plants grown alone. Similarly, the secretion level of chitinase (spot # 84) was higher in the co-culture of Col-Ler compared with other treatments. Finally, the secretion level of another chitinase (spot # 66) was higher in the co-culture of Col-Cap compared with other co-cultures and the plants grown alone (Table S2). Besides these proteins, the majority of the proteins were commonly secreted regardless of the neighbor. For example 68 proteins were commonly secreted between Col, Ler and Col-Ler and 54 proteins were commonly secreted between Col, Cap and Col-Cap. The above data clearly suggest that the root secretion of proteins varied depending upon neighbor identity.

### Novel Proteins Upon Co-culturing of Two Different Individuals

It is noteworthy to mention that the protein exudates collected from the co-cultures are from both plants and that we can’t distinguish from what plant these came from. However, we can distinguish if the co-culture induced the secretion of specific proteins as compared to the plants cultured alone. For instance, one protein [PR5 (spot # 126)] was specifically secreted in the co-culture Col-Ler but not secreted in the co-culture treatments Col-Col and Col-Cap or when the plants (Col, Ler, Cap) were grown alone (Figures 5C & 5D). Similarly, one protein [unknown protein (spot # 123)] was specifically secreted in the co-culture Col-Cap but not in the co-culture treatments Col-Col and Col-Ler or when the plants (Col, Ler, Cap) were grown alone (Figures 5C & 5D).

### Cumulative Levels of Secreted Proteins under Different Functional Categories

**Figure 7:**
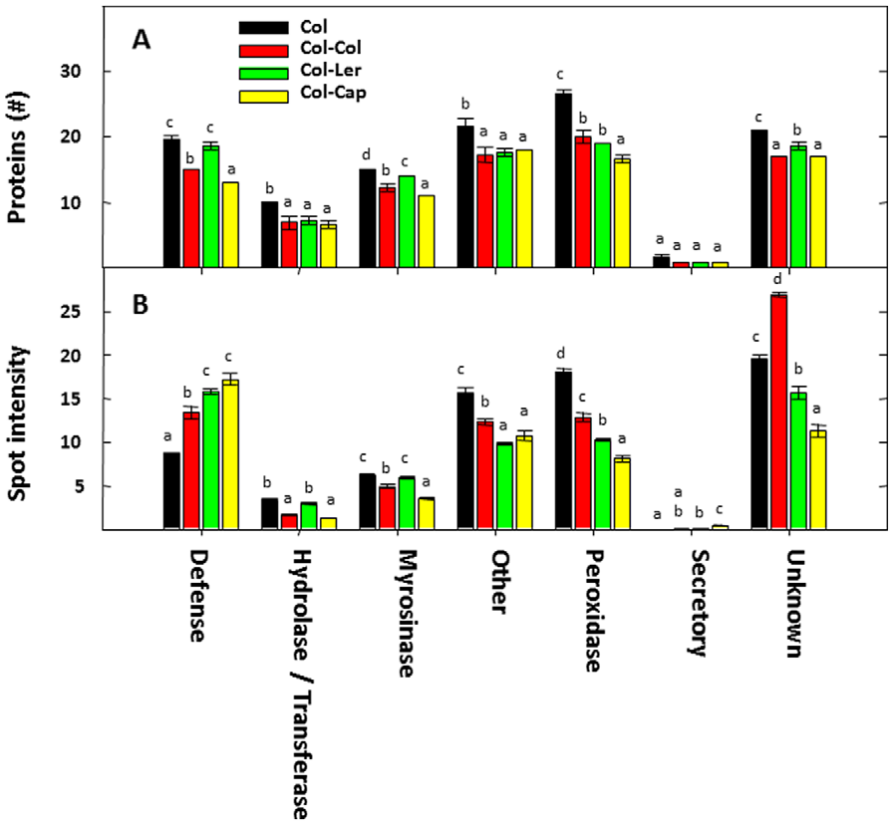
Cumulative analyses of the root secreted proteins of Col, Col-Col, Col-Ler and Col-Cap classified based on their function. (A) Number of proteins in each functional category. (B) Secretion level or cumulative spot intensity of proteins in each functional category. The letters on top of the bars (a, b, c and d) indicate the statistical significance between the treatments compared to each other. The bars with different letters are significantly different (p-value <0.05) from one another.

The dataset was analyzed based on the number of proteins (qualitative) related to each functional category (Figure 7A) and the quantitative accumulation of those proteins (Figure 7B). In general, the number of proteins secreted in each functional category (defense, hydrolases, myrosinases, peroxidases, proteins related to other functional categories and unknown) was higher when Col was grown alone compared with the co-culture treatments (Col-Col, Col-Ler and Col-Cap) (Figure 7A). Interestingly, the number of secretory proteins was low when compared to the other functional categories in all treatments.

The pattern and quantity of overall protein secretion under each functional category varied depending upon the neighbor’s identity with some apparent trends. For example, the total secretion levels of defense proteins increased with the presence of a specific neighbor genotype as compared to the control plant Col grown alone (Col < Col-Col < Col-Ler < Col-Cap) (Figure 7B). On the contrary, the secretion of stress related proteins (peroxidases) decreased with the presence of a specific neighbor genotype as compared to the control plant Col grown alone (Col > Col-Col > Col-Ler > Col-Cap) (Figure 7B).

## Discussion

Competition between plants is a major component of the ecology field and for the most part these studies measure the outcome of the interaction (i.e. biomass, root length, and other visual characteristics) [38], [39]. These studies have largely found that similar species tend to avoid competition and as the level of species un-relatedness increases, competition increases [8], [10], [40]–[42]. Plants tend to compete mainly for water and nutrients in the soil and for light aboveground. In this study, we provided interacting partners with sufficient water and nutrients, and kept the interaction time to a minimum (three days) in an effort to detect the early signals involved in neighbor recognition that might eventually lead to a competitive outcome. In addition, we incubated the co-culture treatments for three days because this minimum secretion time was needed to extract sufficient amount of proteins and metabolites for the metabolomic and proteomic analyses. Therefore, we were not surprised by the lack of proteins involved in nutrient acquisition (i.e. nitrate transporters, sulfur transporters, sugar transporters, other solute transporters, P solubilizing enzymes, etc) in the root exudates of the competing partners because of the following reasons: 1) competition was kept to a minimum time in this study; 2) these proteins are most likely only expressed at the later stages of the competition; and 3) most of these proteins remain in the root tissues and are not necessarily secreted outside the roots. In the present study, our goal was to obtain the first biochemical analysis of the early recognition events between different individuals that might lead to competitive responses.

In the scientific literature, many plant biochemical studies are conducted using single plants in an effort to simplify the experimental conditions. However, our analyses show that the root secreted metabolites and proteins of the single plants are significantly different from that of plants growing with a neighbor. When plants were grown alone, it was observed that they released a higher number of different proteins related to defense, but the cumulative secretion level of these proteins was rather low compared to the co-cultures. Furthermore, the cumulative secretion levels of specific defense related proteins increased depending upon the neighbor identity (Figure 7B). Thus, it seems that a given plant will secrete a large number of defense proteins when grown alone, but once a plant neighbor is identified, the repertoire of proteins will be reduced, but their secretion will be significantly increased. For example, the secretion levels of subtilisin like proteases (spots # 4 & 5) are higher in the co-culture Col-Col compared with other co-culture treatments and the plants grown alone. Similarly, the secretion level of chitinase (spot # 84) was higher in the co-culture of Col-Ler compared with other treatments. Finally, the secretion level of another chitinase (spot # 66) was higher in the co-culture of Col-Cap compared with other co-cultures and the plants grown alone (Table S2). This trend was completely reversed for stress-related proteins where both the number and cumulative secretion of proteins (peroxidases) decreased depending upon the identity of the neighbor. Plant defense-related proteins are largely described in the literature as active against microbial pathogenic infection [43], and peroxidases are usually produced by plants to cope with abiotic and biotic stress [44]–[46]. Similarly, myrosinase binding proteins are involved in defense against fungi, insects and viruses [47], and are induced by wounding and elicited with signaling molecules like jasmonic acid and salicylic acid [48], [49]. To the best of our knowledge, there is not a single report in the literature that has shown that these types of proteins are induced upon the identity of a given plant neighbor.

The present studies suggest that the biochemistry of a single plant might not be the same in field studies involving monocultures of the same plant or polycultures of different plants. A recent study reported that the production of glucosinolates in Arabidopsis leaves was higher in high density of plants grown in pots as compared to low densities [50]. Interestingly, another study has shown that an invasive weed is able to alter its defensive strategies either choosing growth or production of secondary metabolites based on the identity of its plant neighbors [51]. It is also evident in our study that the profile of root secreted secondary metabolites is different from a single plant grown alone vs. co-cultured with homozygous individuals or different individuals.

It is unknown if the different root exudate protein profiles contribute to the sensing and recognition of plant neighbors or whether these are responses to the recognition event. The actual sensing of the neighbor might be based on touch among roots [4] or by earlier chemical signals released by plants, such as the root exudate proteins or metabolites reported in the present study. For example, myrosinase-binding proteins contain several lectin-binding domains, and lectins (e.g., jacalin) are associated with rhizobia-host selectivity [52], [53]. A similar process could be helping plants to differentiate and identify their neighbor. Similarly, peroxidases might also be involved in the process of neighbor recognition as a secondary response followed by recognizing the neighbor through specific receptor proteins or by touch; however, the functions of most of these types of proteins are unknown [46]. Welinder et al. [54] reported that the majority of class III peroxidases are preferentially expressed in the root tissues of Arabidopsis. In addition, our results suggest that specific peroxidases are secreted depending upon the identity of neighbor. Once the neighbor identity has been determined, the number and concentration of defense-related protein secretion varies depending upon the neighbor identity; thus, providing a more selective and targeted response. Additional time-course or mutant studies will be required to prove those hypotheses.

Secondary metabolites have been largely credited to be involved in plant-plant interactions (i.e. allelopathy) with the assumption that these compounds tend to be phytotoxic and persistent in the soil. However, our study showed that the root secreted secondary metabolites could be potentially involved in plant neighbor recognition as it has been demonstrated previously in the context of legume-rhizobia interactions [55]. In contrast, the role of proteins released as root exudates in the soil has been less documented, and proteins present in the soil generally tend to be quickly decomposed by soil microbes, although a small portion are considered to be resistant to microbial decomposition, particularly when associated with mineral and humic substances [56]–[58]. Whether these proteins play a direct phytotoxic role or initiate competitive interactions remains to be determined. However, it is quite evident from our data that some of these proteins are specifically secreted depending on the type of neighbor. For example, a defense protein PR5 was specifically secreted in the co-culture of Col-Ler but absent in the co-cultures of Col-Col or Col-Cap and in the plants Col, Ler and Cap grown alone. Similarly, a putative protein of unknown function was specifically secreted in the co-culture of Col-Cap but absent in the co-cultures of Col-Col or Col-Ler and in the plants Col, Ler, Cap grown alone. These PR5 proteins, called thaumatin-like proteins, are induced by various environmental stresses [59]–[62] and are involved in mediating interactions with their receptors or ligands [43]. These receptors are called PR5-like receptor kinases and in Arabidopsis three PR5-like receptor kinases have been identified [43]. It is likely that these proteins are initiating signal cascades in the other plants that might lead to a subsequent outcome of the competition. Further studies are warranted to identify the candidate signals and signaling pathway mechanisms involved in the early events of plant neighbor recognition.

In this study, we have presented a first glance of root secreted proteins and metabolites involved in the early biochemical events of plant neighbor recognition. The data set presented here suggest that the early events of plant competition involve more than nutrient sensing and these events also include a biochemical realization that a given plant is interacting with a similar or different individual. The realization of differences among interacting plant neighbors is complex and, in part, involves a differentiation in both the quantity and identity of proteins in the root exudates, which appears to be influenced by the level of relatedness to its neighbor. The implications of this study are multipronged but it seems that the understanding of the ways that crops behave in monocultures vs. polycultures will allow us to device strategies by which the plants could better defend themselves.

## Supporting Information

Figure S1 **A representative two-dimensional proteomic map of the total secreted proteins of the plants grown alone, co-cultured with the homozygous individual and co-cultured with the different individuals.** The molecular masses (kDa) of protein standards are indicated to the left side of the gel and the isoelectric point (pI) is indicated at the top of the gel. The arrows with numbers represent the proteins identified which are listed in Table S1.
(TIF)

Figure S2 **Diagram depicting the total number of proteins identified in the 2-D gels of each treatment analyzed by PDQuest 8.0.1.** Different treatment names are shown inside the boxes and the numbers represented on top of each box account for the total number of protein spots identified. Numbers represented next to the lines are the number of proteins commonly shared between the two treatments.
(TIF)

Table S1 **List of all root secreted metabolites mass features and their abundance values from all the experimental conditions.**
(PDF)

Table S2 **List of all root secreted proteins and their abundance values (spot intensity) from all the experimental conditions.**
(PDF)

Table S3 **Univariate ANOVA comparing each individual protein across treatments.**
(PDF)

Table S4 **Total secreted proteins by category of individually grown or plants co-cultured with homologous or different individuals.**
(PDF)

Table S5 **Univariate ANOVA comparing each protein category across treatments.**
(PDF)

Table S6 **Multivariate ANOVA comparing protein profiles by category across treatments.**
(PDF)
