# Supplementary material for: Root Secreted Metabolites and Proteins Are Involved in the Early Events of Plant-Plant Recognition Prior to Competition
Source: PLoS One. 2012 Oct 2;7(10):e46640. doi: 10.1371/journal.pone.0046640 (PMC3462798; doi:10.1371/journal.pone.0046640)
Supplement: Table S1 — List of all root secreted metabolites mass features and their abundance values from all the experimental conditions. (PDF) [file pone.0046640.s003.pdf]

Table S1. List of root secreted metabolites (mass features) in individually grown or plants co-cultured with homologous or different individuals. .
The values represent the relative ion intensity of each mass trace. All reported values are the arithmetic mean (n = 3) of the relative ion intensity values and standard error (SE)

Mass (H+)	Retention time (min)	Col	SE	Col-Col	SE	Col-Ler	SE	Col-Cap	SE	Ler	SE	Ler-Ler	SE	Cap	SE	Cap-Cap	SE
181.15	3.90	0	0	0	0	0	0	0	0	1091559	99343	0	0	0	0	0	0
167.17	4.08	0	0	0	0	0	0	0	0	0	0	5645901	242885	0	0	3418163.9	455944
193.12	4.12	0	0	8241606	619930	0	0	0	0	0	0	8767257	75258	0	0	0	0
459.15	4.68	0	0	543712	28488	385995	23332	466849	24786	0	0	0	0	0	0	0	0
203.10	4.71	0	0	1784873	146257	0	0	0	0	2085010	382444	1608457	152235	1275802	43428	0	0
233.10	5.86	0	0	1895495	172061	2036709	323522	1570057	28665	0	0	0	0	0	0	0	0
192.23	14.31	0	0	1788978	260303	1289164	45857	17209254	2111696	0	0	0	0	0	0	0	0
504.19	19.66	0	0	53563	4434	0	0	0	0	0	0	516031	51730	0	0	8433	4493
629.24	20.66	0	0	35481	5905	0	0	0	0	0	0	249092	24349	0	0	1978	571
178.17	22.98	0	0	2214067	361455	793358	242212	0	0	0	0	2311513	296800	0	0	0	0
173.23	24.50	0	0	833434	108371	415245	25485	933634	55648	0	0	0	0	0	0	0	0
188.22	25.18	0	0	4975233	553208	0	0	4228564	657320	0	0	0	0	0	0	0	0
209.18	25.76	0	0	13808108	679066	0	0	17238454	2161714	0	0	0	0	0	0	0	0
247.12	25.90	0	0	2601501	314759	2234877	287926	4894222	267848	0	0	5902003	570150	0	0	0	0
179.20	26.10	0	0	2728158	201099	1222856	119695	1868083	199095	0	0	0	0	0	0	0	0
177.25	27.28	0	0	188424	18497	0	0	0	0	0	0	599763	52382	0	0	243942	40019
176.25	27.41	0	0	418206	49801	0	0	0	0	0	0	1045069	179971	0	0	0	0
194.23	27.42	0	0	562588	53831	0	0	0	0	0	0	2375484	254643	0	0	257320	56772
387.17	27.81	0	0	20783779	1016370	16111701	2688354	36247638	10795100	0	0	0	0	0	0	0	0
389.18	27.86	0	0	1422935	268376	1137147	161591	1891533	295465	0	0	2033645	367059	0	0	2230408	553928
472.29	27.96	0	0	1855377	232194	1155522	213809	1077116	92708	0	0	1628013	203039	0	0	173319	71330
471.31	28.08	0	0	10068401	1243687	6627509	1529693	4618424	660861	0	0	8641263	1081431	0	0	1008551	401487
441.23	28.80	4109596	129775	4919750	244718	3436395	77077	0	0	1251078	290569	0	0	0	0	0	0
120.23	28.87	0	0	0	0	0	0	0	0	0	0	37944521	4270506	0	0	24992677	4270949
436.26	28.94	6520650	866042	0	0	0	0	0	0	0	0	0	0	53319	7736	0	0
727.49	28.99	6219292	300520	5460156	1195484	1787542	589407	6334681	715692	386815	120320	4950614	226131	28435	2283	34897	13335
617.44	29.03	0	0	13603961	2649887	0	0	0	0	236247	119114	19458897	992417	3040845	951330	13553734	3658611
926.75	29.06	0	0	395512	52748	232021	49345	0	0	0	0	501046	36537	0	0	0	0
949.65	29.08	0	0	36816	3511	0	0	0	0	0	0	43126	2423	0	0	59610	14102
877.59	29.11	0	0	0	0	0	0	162074	18997	0	0	0	0	0	0	0	0
904.56	29.27	0	0	72048	12706	0	0	0	0	0	0	249843	51074	0	0	8249	2081
1041.69	29.31	0	0	0	0	0	0	0	0	0	0	577	167	0	0	51590	15066
878.55	29.40	2236.19	476.035	0	0	0	0	0	0	0	0	0	0	211056	14110	0	0
310.35	29.49	0	0	0	0	0	0	0	0	18868625	7957386	0	0	49836160	3939644	0	0
510.36	29.50	0	0	666532	131525	0	0	0	0	0	0	3194047	532658	0	0	69400	19796
741.50	29.62	29516.1	6542.34	0	0	0	0	0	0	18715	10709	0	0	341905	30985	0	0
925.77	29.64	0	0	0	0	0	0	0	0	55624	27101	0	0	745279	154653	0	0
202.18	29.69	0	0	18141723	2995403	0	0	0	0	0	0	45725310	7181001	0	0	894591	191801
560.33	29.77	0	0	155975	18096	0	0	0	0	0	0	1060897	134946	0	0	0	0
743.53	29.80	8069.39	2514.44	0	0	0	0	0	0	0	0	0	0	477295	62990	0	0
899.53	29.82	0	0	0	0	0	0	0	0	0	0	278275	45059	0	0	41983	12309
204.18	29.88	0	0	7114539	314517	0	0	0	0	0	0	19712329	1438358	0	0	336261	23772
142.28	29.89	8241562	1688406	0	0	0	0	0	0	0	0	0	0	37647833	2296481	0	0
745.52	29.91	7625.07	2066.59	0	0	0	0	0	0	0	0	0	0	184686	8284	0	0
205.23	29.94	0	0	1255401	63920	1188963	269897	2194301	110336	0	0	0	0	0	0	0	0
192.17	30.62	1458866	74248.8	0	0	0	0	0	0	0	0	0	0	21518	12424	0	0
323.18	30.87	0	0	13298123	428767	11455614	2323839	13261496	2346034	0	0	30196136	5034872	0	0	0	0

579.25	31.29	0	0	2484659	160181	1466267	75018	0	0	0	0	3663129	518807	0	0	602038	153461
580.27	31.31	0	0	626039	47905	406420	53642	750316	59816	0	0	0	0	0	0	0	0
433.19	31.45	0	0	675035	74055	421751	61093	832301	116727	0	0	0	0	0	0	0	0
808.52	31.57	0	0	157655	15558	0	0	0	0	0	0	1297248	187045	0	0	81300	23180
183.22	32.02	0	0	0	0	0	0	0	0	273608	54637	0	0	1260901	163843	0	0
266.20	33.42	0	0	1666782	221168	4801113	490288	569091	239275	0	0	0	0	0	0	0	0
716.42	33.46	0	0	186089	42491	0	0	314603	39653	0	0	868697	60672	0	0	0	0
288.16	33.53	0	0	0	0	0	0	0	0	4647694	1664104	0	0	0	0	0	0
895.66	33.83	0	0	0	0	1668	341	3622	363	0	0	0	0	0	0	0	0
198.24	35.01	0	0	0	0	2384105	409068	5219342	873557	0	0	0	0	0	0	0	0
496.28	35.94	0	0	109166	16783	0	0	0	0	0	0	322913	26445	0	0	193581	39993
546.22	36.08	1100641	87314.4	0	0	0	0	0	0	143196	62325	0	0	4984	2877	0	0
540.28	36.11	0	0	4549019	1013188	1520390	399013	6096950	436592	0	0	0	0	0	0	0	0
541.26	36.15	2170719	217618	1029404	143057	0	0	0	0	0	0	514439	72386	45629	1521	106276	30597
545.21	36.17	0	0	2207055	292003	1130636	224017	1388827	191397	0	0	0	0	0	0	0	0
219.18	37.53	0	0	3415683	426103	0	0	0	0	0	0	15160270	2186436	0	0	4745588	1271954
357.20	37.57	0	0	19361945	1512599	0	0	0	0	0	0	37084144	6225133	0	0	1159810	201876
730.49	37.58	0	0	334280	26868	121254	43033	0	0	0	0	545279	47207	0	0	15642	3987
731.48	37.66	0	0	206602	27539	0	0	0	0	0	0	313667	20141	0	0	0	0
357.19	37.99	0	0	7253941	534761	4698990	793001	0	0	0	0	13105483	462508	0	0	322502	96653
390.33	38.36	0	0	1792330	128331	0	0	0	0	0	0	1886135	409245	0	0	112209	24465
244.22	39.10	0	0	0	0	0	0	7008395	582521	0	0	0	0	0	0	0	0
192.23	39.14	0	0	0	0	1260415	58989	1709859	121856	0	0	0	0	0	0	0	0
373.30	39.34	0	0	2139439	228608	0	0	0	0	0	0	1565132	235596	0	0	110610	23279
201.12	40.49	0	0	30939307	8467521	19155542	2858203	57633694	10892480	0	0	0	0	0	0	0	0
234.16	40.88	0	0	72012053	4271459	0	0	0	0	0	0	114965707	7949404	0	0	1001342	238157
198.26	42.55	0	0	1431756	400720	1833747	152058	2078181	220730	0	0	783721	86660	0	0	1751254	197399
500.43	43.30	0	0	4440575	509744	0	0	0	0	0	0	12512317	1308187	0	0	8141510	2260430
475.35	43.38	1120000	110396	0	0	0	0	0	0	243179	39826	0	0	0	0	0	0
501.39	43.62	0	0	0	0	0	0	0	0	488251	174635	0	0	2863510	797049	0	0
371.31	43.64	0	0	13976618	2672705	7653881	1527508	731223	148016	0	0	16674491	2385742	0	0	0	0
484.43	44.02	0	0	656962	3888	0	0	0	0	0	0	2013936	169555	0	0	689988	177117
559.49	45.14	0	0	2396556	383178	0	0	0	0	0	0	5988086	982463	0	0	3210753	933805
408.41	45.59	0	0	382106	16677	247122	46945	391225	59628	0	0	0	0	0	0	0	0
430.38	45.76	3253538	382991	2445633	4470	1844491	128164	1088084	265919	0	0	3760823	579216	0	0	443357	120397
473.34	45.95	0	0	437896	91937	441650	46235	746496	22434	0	0	0	0	0	0	0	0
621.50	46.65	0	0	0	0	0	0	0	0	0	0	6579906	673635	0	0	1490036	598441
943.70	47.22	0	0	0	0	0	0	0	0	6743	3011	0	0	54098	16124	0	0
487.37	47.38	0	0	13210949	1330400	7973350	808983	13083029	3701379	0	0	32733279	3517505	0	0	4740053	880922
482.43	47.44	0	0	20256018	2581068	4069935	185215	8676595	2725927	0	0	56969708	5458954	0	0	0	0
943.75	47.60	0	0	0	0	0	0	0	0	1312658	697768	0	0	7105021	1836460	0	0
674.55	47.82	0	0	36155644	2854719	0	0	0	0	0	0	40708705	15856434	0	0	10854481	2787860
679.49	47.89	0	0	11429401	766833	4157445	814156	2617177	673211	0	0	9332293	1618790	0	0	2767507	699518
676.59	47.91	0	0	8101237	685899	0	0	0	0	0	0	2572568	386390	0	0	732099	199102
746.68	48.03	0	0	241793	16284	104036	16610	165905	42367	0	0	0	0	0	0	0	0
680.55	48.04	0	0	2555695	321739	1498335	230462	1204703	247289	380839	27889	2550974	368952	508526	46837	0	0
808.77	48.36	0	0	0	0	0	0	0	0	10754	5783	0	0	909451	357200	0	0
658.49	48.53	0	0	1911576	188154	1040240	116846	703508	110658	0	0	2123737	714931	0	0	0	0
663.46	48.58	0	0	2652971	322862	1956033	418689	925507	209161	0	0	3340969	430272	0	0	0	0
545.44	48.64	0	0	19865336	1943225	10242294	1027105	14685482	3328873	0	0	26450965	746448	0	0	0	0
540.48	48.65	0	0	45973131	4856160	34757541	4827948	53303198	13831904	0	0	111046036	4836164	0	0	0	0

541.48	48.79	0	0	0	0	4756718	186348	11846154	2642922	0	0	0	0	0	0	0	0
739.65	48.92	0	0	382822	31518	0	0	0	0	0	0	664527	162186	0	0	349127	77148
702.58	48.94	0	0	1387296	315894	542349	140990	246460	53235	0	0	1071310	331983	0	0	0	0
733.70	49.00	2092044	173976	5046259	329240	2326265	415045	3057727	417662	1203482	535321	0	0	5529355	2274051	0	0
738.67	49.01	0	0	2057897	251673	1253036	161342	1109603	198077	0	0	2847416	530509	0	0	0	0
737.69	49.03	0	0	6020531	355981	3636237	159405	3332139	839435	3105764	1002299	0	0	5493976	1683824	0	0
732.71	49.05	1.1E+07	1302330	12143390	683349	6192597	526599	8168882	2151283	2068974	756854	0	0	13315708	4072551	0	0
734.68	49.07	0	0	0	0	0	0	0	0	141897	45614	0	0	674934	126636	0	0
708.58	49.15	0	0	367172	36886	209333	51385	50102	8774	0	0	163772	48475	0	0	0	0
806.83	49.23	0	0	204837	15040	84348	9612	108852	26907	0	0	395250	86140	0	0	0	0
692.53	49.37	0	0	1345826	111532	707591	23222	280229	32420	0	0	604060	35905	0	0	483683	66062
181.14	49.38	0	0	0	0	0	0	0	0	22042661	4892394	14271026	1011352	15932477	2852030	9510582	976045
301.21	49.41	0	0	19292814	943993	12166098	1611720	11139711	957382	0	0	0	0	0	0	0	0
691.48	49.42	0	0	3457334	249192	0	0	0	0	0	0	1866953	116612	0	0	460282	63888
580.39	49.43	0	0	668483	49227	560581	55991	585695	35635	684768	108561	586044	101360	423453	56563	0	0
596.50	49.46	0	0	296271	6254	0	0	0	0	0	0	1052844	149766	0	0	0	0
182.19	49.47	0	0	1791167	104045	1683544	355144	1470032	224664	0	0	0	0	0	0	0	0
195.13	49.50	0	0	0	0	0	0	0	0	4348397	913916	0	0	4384751	618195	0	0
279.24	49.53	0	0	4036780	324290	2921975	45522	0	0	7050064	1631208	0	0	6852303	1122717	0	0
399.34	49.67	0	0	4484711	204397	4241367	57579	4889499	388250	7344218	1516700	5264152	181221	14244007	454914	0	0
721.61	49.68	0	0	1147574	200522	550528	70204	0	0	0	0	0	0	0	0	0	0
812.72	49.84	0	0	0	0	0	0	0	0	58166	15947	0	0	70906	7338	0	0
795.72	49.85	0	0	0	0	0	0	0	0	0	0	804798	68049	0	0	1302506	310632
599.58	49.88	0	0	16188598	724645	6361299	282416	0	0	0	0	0	0	0	0	0	0
603.52	49.90	0	0	29280753	4557720	27104059	4705709	7739091	1728452	0	0	53717627	3692781	0	0	0	0
598.56	49.94	0	0	109228506	7548662	31082651	6914497	33465825	9393207	0	0	0	0	0	0	0	0
599.55	49.98	0	0	22678507	813565	8847437	1359914	12338890	3010986	0	0	0	0	0	0	0	0
792.78	50.08	0	0	469718	29022	240700	31364	451178	172618	0	0	661983	194372	0	0	0	0
792.74	50.14	0	0	869780	63024	0	0	0	0	0	0	1340552	230042	0	0	1068588	329071
760.73	50.15	0	0	1454465	252929	704434	100899	226634	45229	0	0	1027358	330918	0	0	0	0
399.30	50.16	0	0	0	0	0	0	0	0	1541420	295245	0	0	2737752	351076	0	0
791.81	50.20	0	0	2039959	112243	1178773	182934	0	0	0	0	0	0	0	0	0	0
851.82	50.65	0	0	68796	21306	0	0	0	0	0	0	259341	79084	0	0	99172	35638
846.81	50.72	0	0	235327	54988	90274	11864	191893	35762	0	0	549175	128547	0	0	270214	101711
745.66	50.76	0	0	118592	2395	78856	24010	45093	12969	0	0	0	0	0	0	0	0
775.75	50.80	0	0	798559	102953	454580	42750	957267	300445	0	0	1156964	254240	0	0	0	0
849.85	51.07	0	0	1636833	246021	2590112	491317	6922781	2055879	0	0	6412810	1077543	0	0	0	0
661.52	51.12	0	0	54277608	10146513	37643947	6753478	22143220	6306716	0	0	69988517	11173565	0	0	0	0
850.83	51.21	0	0	194669	31446	187699	24771	700299	222587	0	0	0	0	0	0	0	0
658.59	51.22	0	0	3257430	229498	1674455	176269	0	0	0	0	0	0	0	0	0	0
639.56	51.24	0	0	1986707	211283	1168199	206099	1172751	386012	0	0	1278183	479756	0	0	0	0
853.78	51.30	3169777	230170	0	0	0	0	0	0	895479	246326	981703	161690	1675880	540485	1149833	207609
657.58	51.31	0	0	20657270	896657	10026932	2061715	0	0	0	0	0	0	0	0	0	0
841.80	51.65	0	0	514629	145209	342360	60548	539479	89668	0	0	338155	74361	0	0	0	0
753.70	51.73	0	0	1137401	180987	645732	77219	1342308	206045	0	0	0	0	0	0	0	0
886.77	51.74	0	0	0	0	0	0	0	0	37762	16441	0	0	190295	47333	0	0
884.81	51.74	0	0	0	0	0	0	0	0	870922	314922	0	0	1519012	374681	0	0
840.77	51.75	0	0	0	0	0	0	0	0	804062	348551	0	0	2773758	573343	0	0
752.74	51.76	0	0	2692079	529015	1611882	205733	4811757	437100	0	0	2803855	246628	0	0	0	0
796.77	51.77	0	0	1797491	401022	1735680	271833	1898694	385926	1818575	363923	1482101	254353	3111525	311762	0	0
489.41	51.83	0	0	803671	53005	726040	115622	1206979	152823	0	0	1433640	141512	0	0	0	0

620.55	51.83	0	0	12420290	1990775	12917132	2254221	11306887	1490414	0	0	17780004	3413489	0	0	11033444	2888619
752.71	51.85	0	0	2579428	487184	2105350	172171	0	0	5355570	1809998	2326656	309419	11253635	2345687	3871730	897363
709.61	51.89	0	0	1919798	227045	0	0	2851683	258702	1468850	288800	0	0	5303690	1529397	0	0
798.73	51.89	0	0	0	0	0	0	0	0	177058	77693	0	0	424563	74835	0	0
708.60	51.91	0	0	5883081	1580840	5758907	1177984	6820364	869967	5740577	909932	5711701	569798	18275861	3718423	5960235	943729
621.55	51.91	0	0	4843899	503244	0	0	0	0	5539129	1457634	3919576	606214	7349349	1007518	4041309	872866
576.50	51.96	0	0	17551696	3230412	16446058	1131093	22608777	1018573	0	0	15665670	2289878	0	0	16443852	1301032
753.66	51.98	0	0	1470698	326730	845502	173081	2063649	259642	0	0	0	0	0	0	0	0
664.53	51.98	0	0	5791184	1343063	0	0	6763001	1176835	9196628	1950818	0	0	29936459	6123733	0	0
577.51	51.99	0	0	3794459	1002516	3093563	296612	6998055	610843	4103315	1056878	7220593	643134	4524044	213462	0	0
533.44	51.99	0	0	3654646	266014	2013479	90161	3591040	736217	3503304	839313	0	0	4538421	905715	0	0
622.52	51.99	0	0	0	0	0	0	0	0	563108	110410	0	0	887239	224094	0	0
532.41	51.99	0	0	0	0	0	0	0	0	4626523	1058665	0	0	4285631	979489	0	0
488.42	52.03	0	0	3375556	233824	3605777	168827	4715946	196100	0	0	4872177	485455	0	0	0	0
538.38	52.06	0	0	0	0	0	0	0	0	965802	86977	0	0	754471	100301	0	0
581.44	52.09	0	0	2148590	118939	1719868	168406	1565120	146501	2915939	281368	0	0	2064159	520531	0	0
494.36	52.09	0	0	0	0	0	0	0	0	1007272	148792	0	0	785551	88896	0	0
582.45	52.10	0	0	520271	71633	0	0	0	0	0	0	359328	52623	0	0	286887	39072
904.86	52.23	0	0	0	0	0	0	0	0	83495	44324	0	0	259792	83530	0	0
911.85	52.38	0	0	0	0	0	0	0	0	0	0	711284	96847	0	0	1075280	264319
721.62	52.69	0	0	3240281	421639	1507202	249688	1559841	530471	0	0	0	0	0	0	0	0
714.66	52.72	0	0	38164164	2210457	0	0	0	0	0	0	53825593	11855191	0	0	27458962	8209087
750.69	53.16	0	0	421824	160721	374884	109137	406546	73245	0	0	359139	103111	0	0	0	0
891.85	53.40	0	0	195086	9964	180384	42870	0	0	0	0	0	0	0	0	0	0
965.97	53.84	0	0	114868	7823	0	0	0	0	0	0	512390	225611	0	0	568791	15364
964.96	53.85	0	0	500521	45759	0	0	0	0	0	0	1435987	233807	0	0	2095556	205435
772.77	54.26	0	0	0	0	0	0	0	0	11002596	4902318	0	0	25305935	12543653	0	0
779.73	54.29	0	0	1404832	122410	0	0	0	0	0	0	1634711	367361	0	0	1379990	416099
986.93	54.54	0	0	0	0	0	0	0	0	0	0	0	0	0	0	678926	82160
944.83	54.55	0	0	64204	18810	0	0	0	0	0	0	198953	35080	0	0	239906	19886
1030.99	54.55	0	0	354420	96917	261399	87202	519838	74595	0	0	0	0	0	0	0	0
856.76	54.71	0	0	361878	71524	201570	26960	465688	61459	120890	24257	0	0	197621	63768	0	0
766.73	55.07	0	0	0	0	1390880	385168	4547743	1219706	0	0	0	0	0	0	0	0
898.84	55.10	0	0	0	0	0	0	0	0	453161	91403	0	0	933913	231803	0	0
811.77	55.13	0	0	0	0	0	0	0	0	1002941	189856	0	0	1226618	335532	0	0
458.45	55.18	0	0	799201	91274	705660	37098	1095670	43876	0	0	947352	79243	0	0	1027062	85564
636.57	55.24	0	0	0	0	350293	56684	992961	334864	0	0	0	0	0	0	0	0
590.53	55.28	0	0	5664215	666513	3250752	472056	8336888	2205390	0	0	0	0	0	0	0	0
552.43	55.34	0	0	457867	38601	191978	26264	0	0	0	0	442801	68201	0	0	0	0
547.46	55.50	0	0	2372429	364058	0	0	0	0	0	0	1621732	110116	0	0	0	0
831.85	55.74	0	0	1883298	243550	3848297	1052392	4181476	1453895	0	0	12348441	2675172	0	0	2503238	726027
659.35	56.33	0	0	0	0	0	0	0	0	0	0	3427400	390071	0	0	2517725	71283
654.41	56.35	0	0	34782879	7799700	34936941	1126444	37201254	5018211	12068943	3557156	47350481	6495437	50748429	927480	54423128	6624022
637.40	56.38	0	0	39317938	2340269	15397477	587812	31710565	5938365	15978988	5540751	32744642	4365185	35670101	903551	16135634	2310802
670.42	56.42	0	0	2475771	213395	1685268	112262	2842008	562425	0	0	4001242	138632	0	0	0	0
656.44	56.45	287901	81946.2	2097982	136333	1298446	88827	0	0	320331	98802	1412226	35486	1078961	160091	0	0
638.44	56.48	0	0	4458665	432430	2913404	169608	4942016	317513	2220772	504667	0	0	2392150	163054	0	0
1103.79	56.59	0	0	19891	2493	0	0	0	0	0	0	25201	5668	0	0	0	0
1190.99	56.61	0	0	36263	3337	119636	33625	194804	18534	0	0	0	0	0	0	0	0
1190.00	56.62	0	0	18552	3318	170730	29398	223639	39300	0	0	240666	9544	0	0	0	0
889.82	57.95	0	0	0	0	0	0	0	0	0	0	0	0	0	0	5111236	1253771
