# Supplementary material for: Root Secreted Metabolites and Proteins Are Involved in the Early Events of Plant-Plant Recognition Prior to Competition
Source: PLoS One. 2012 Oct 2;7(10):e46640. doi: 10.1371/journal.pone.0046640 (PMC3462798; doi:10.1371/journal.pone.0046640)
Supplement: Table S2 — List of all root secreted proteins and their abundance values (spot intensity) from all the experimental conditions. (PDF) [file pone.0046640.s004.pdf]

Table S2. List of secreted proteins in individually grown or plants co-cultured with homologous or different individuals.
All reported values are the arithmetic mean (n = 3) and standard deviation.

							Protein Spot Intensity							
**Spot #**	**Protein ID**	**Accession**	**Score**	**Peptides**	**pI**	**MW**	**Col**	**Col-Col**	**Col-Ler**	**Col-Cap**	**Ler**	**Ler-Ler**	**Cap**	**Cap-Cap**
**Myrosinases**														
1	putative myrosinase-binding protein	AAD12681	590	10	5.5	32672	1.04 (0.121)	0.16 (0.034)	0.698 (0.019)	0.474 (0.034)	0.038 (0.007)	0.185 (0.03)	0 (0)	0 (0)
2	putative myrosinase-binding protein	AAD12681	253	5	5.5	32672	0.9 (0.132)	0.69 (0.105)	0.574 (0.02)	0.317 (0.012)	0 (0)	0.196 (0.024)	0 (0)	0 (0)
27	myrosinase-associated protein	AAM64922	177	6	6.7	43042	0.098 (0.001)	0.031 (0.004)	0 (0)	0 (0)	0 (0)	0 (0)	0.054 (0.01)	0 (0)
31	putative myrosinase-binding protein	AAD12681	62	2	5.5	32672	0.245 (0.009)	0.389 (0.032)	0.012 (0.002)	0.151 (0.006)	0.054 (0.006)	0.01 (0.008)	0.046 (0.016)	0.041 (0.011)
42	putative myrosinase-binding protein	AAD12681	417	7	5.5	32672	0.533 (0.012)	0.87 (0.078)	0.506 (0.02)	0.395 (0.021)	1.365 (0.186)	0.766 (0.053)	0.359 (0.034)	0 (0)
58	Similar to gi|3413714 T19L18.21 putative myrosinase-binding prot	AAD12681	68	2	5.5	32672	0.284 (0.012)	0.011 (0.019)	0.09 (0.026)	0.4 (0.21)	0.333 (0.063)	0.378 (0.039)	0.21 (0.024)	0.233 (0.045)
92	Similar to gi|3413714 T19L18.21 putative myrosinase-binding prot	AAD12679	91	1	5.8	32309	0.164 (0.017)	0 (0)	0 (0)	0.088 (0.007)	0.599 (0.08)	0 (0)	0.029 (0.011)	0 (0)
97	Similar to gi|3413714 T19L18.21 putative myrosinase-binding prot	AAD12679	91	1	5.8	32309	0.017 (0.01)	0 (0)	0.117 (0.018)	0 (0)	0.138 (0.01)	0.171 (0.015)	0 (0)	0 (0)
98	Similar to gi|3413714 T19L18.21 putative myrosinase-binding prot	AAD12679	125	2	5.8	32309	0.301 (0.013)	0.382 (0.038)	0.191 (0.049)	0 (0)	0 (0)	0.041 (0.009)	0 (0)	0 (0)
99	Similar to gi|3413714 T19L18.21 putative myrosinase-binding prot	AAD12679	91	1	5.8	32309	0.046 (0.011)	0.279 (0.033)	1.105 (0.127)	0.05 (0.016)	0.432 (0.194)	0.451 (0.06)	0 (0)	0 (0)
101	putative myrosinase-binding protein	AAD12679	260	4	5.8	32309	0.035 (0.008)	0.279 (0.056)	0.116 (0.031)	0.232 (0.024)	0.699 (0.092)	0.73 (0.282)	0 (0)	0 (0)
104	putative myrosinase-binding protein	AAD12679	306	6	5.8	32309	2.38 (0.186)	1.184 (0.151)	0.951 (0.044)	0.784 (0.056)	0.699 (0.171)	0.198 (0.022)	1.56 (0.453)	2.667 (0.598)
105	putative myrosinase-binding protein	AAD12679	170	3	5.8	32309	0.099 (0.021)	0.321 (0.012)	0 (0)	0.497 (0.007)	0.022 (0.007)	0.607 (0.028)	1.031 (0.042)	0 (0)
107	putative myrosinase-binding protein	AAD12681	222	3	5.5	32672	0.036 (0.004)	0.179 (0.007)	0.515 (0.038)	0.195 (0.022)	0 (0)	0.162 (0.051)	0.008 (0.006)	0.404 (0.031)
108	putative myrosinase-binding protein	AAD12681	126	3	5.5	32672	0.014 (0.002)	0.179 (0.001)	0.375 (0.02)	0 (0)	0.041 (0.035)	0 (0)	0 (0)	0 (0)
125	putative myrosinase-binding protein	AAD12679	125	2	5.8	32309	0 (0)	0 (0)	0.471 (0.014)	0 (0)	0.597 (0.103)	0.399 (0.048)	0 (0)	0 (0)
127	Similar to gi|3413714 T19L18.21 putative myrosinase-binding prot	AAD12679	181	3	5.8	32309	0 (0)	0 (0)	0.264 (0.018)	0 (0)	0.407 (0.029)	0.431 (0.042)	0 (0)	0 (0)
**Defense-related proteins**														
4	subtilisin-like protease	CAA59963	125	2	5.8	78164	0.043 (0.004)	0.184 (0.039)	0.025 (0.027)	0.007 (0.003)	0.009 (0.007)	0 (0)	0.004 (0.004)	0 (0)
5	subtilisin-like protease	CAA59963	125	2	5.8	78164	0.225 (0.067)	0.446 (0.104)	0.143 (0.004)	0.128 (0.015)	0.088 (0.007)	0.019 (0.012)	0 (0)	0.004 (0.004)
30	alpha-galactosidase	NP_196455	255	5	6.1	45680	0.167 (0.046)	0.221 (0.034)	0.029 (0.004)	0.125 (0.011)	0.033 (0.003)	0 (0)	0.016 (0.012)	0.017 (0.005)
38	BGL2 (PATHOGENESIS-RELATED PROTEIN 2);	NP_191285	158	3	4.9	37316	0.25 (0.02)	0.12 (0.026)	0.016 (0.002)	0 (0)	0 (0)	0.122 (0.042)	0 (0)	0 (0)
45	beta-1,3-glucanase-like protein	BAB08587	145	3	5.7	50717	0.558 (0.046)	1.171 (0.727)	0.149 (0.01)	0.881 (0.042)	0.155 (0.068)	0.043 (0.039)	0 (0)	0.134 (0.03)
46	subtilisin-like protease	CAA59963	70	3	5.8	78164	0.085 (0.001)	0 (0)	0 (0)	0 (0)	0.04 (0.015)	0.019 (0.003)	0.016 (0.008)	0 (0)
54	subtilisin-like protease	CAA59963	70	3	5.8	78164	0.055 (0.01)	0 (0)	0.032 (0.006)	0 (0)	0.061 (0.011)	0 (0)	0.012 (0.007)	0 (0)
57	AT4g16260/dl4170c (beta-1,3-glucanase class I precursor)	AAL36038	188	4	6.4	37688	0.086 (0.001)	0 (0)	0.348 (0.026)	0.014 (0.002)	0 (0)	0 (0)	0 (0)	0 (0)
62	glucan 1,3-beta-glucosidase	NP_191285	412	9	4.9	37316	0.001 (0.001)	0.486 (0.092)	1.085 (0.168)	0.211 (0.015)	0 (0)	0.142 (0.035)	1.12 (0.298)	0 (0)
64	BGL2	NP_191285	295	5	4.9	37316	0.527 (0.025)	0.333 (0.039)	1.085 (0.014)	0.388 (0.051)	1.517 (0.318)	1.519 (0.421)	1.195 (0.033)	1.616 (0.198)
65	BGL2	NP_191285	295	5	4.9	37316	0.041 (0.006)	0.231 (0.044)	1.085 (0.045)	0.936 (0.108)	1.45 (0.245)	0.89 (0.122)	0.454 (0.061)	0.454 (0.23)
66	chitinase	NP_181887	70	2	8.4	28334	4.633 (0.133)	5.283 (0.383)	3.586 (0.266)	7.776 (0.47)	4.064 (0.332)	1.21 (0.167)	3.358 (0.386)	3.098 (0.093)
75	basic chitinase	1710349A	173	3	8.1	36091	0.023 (0.006)	0.805 (0.043)	0.078 (0.005)	0 (0)	0.267 (0.047)	0.368 (0.054)	3.024 (1.116)	0.509 (0.066)
80	chitinase	NP_181885	141	2	5.8	29756	0.453 (0.018)	0.689 (0.014)	0.809 (0.009)	0 (0)	2.097 (0.305)	0 (0)	0 (0)	1.124 (0.209)
83	class I chitinase	AAF69780	173	3	7.5	34259	0.122 (0.033)	0 (0)	0 (0)	0 (0)	0.112 (0.041)	0.047 (0.029)	0 (0)	0.027 (0.006)
84	chitinase	NP_181885	157	3	5.8	29756	0.261 (0.033)	0 (0)	0.808 (0.019)	0 (0)	0 (0)	0.217 (0.038)	0.237 (0.037)	0.112 (0.033)
86	PR5	NP_177641	186	3	4.8	25236	0.043 (0.001)	0.605 (0.074)	1.521 (0.055)	0.303 (0.012)	0.371 (0.043)	0.871 (0.111)	0.12 (0.1)	0 (0)
88	chitinase	NP_181887	137	2	8.4	28334	0.499 (0.012)	1.014 (0.171)	0.947 (0.048)	4.394 (0.268)	2.999 (0.893)	0 (0)	0.738 (0.129)	1.197 (0.197)
91	Osmotin	CAA61411	236	4	6.7	26585	0.005 (0.001)	0.11 (0.026)	0.1 (0.006)	0 (0)	0.413 (0.256)	0.792 (0.104)	0.295 (0.021)	0.103 (0.016)
113	Pathogenesis related PR-1 protein	NP_179068	110	2	9.1	17666	0.69 (0.052)	1.69 (0.058)	3.66 (0.393)	0 (0)	6.82 (1.067)	3.567 (0.456)	0.215 (0.051)	1.144 (0.338)
118	PR5 (PATHOGENESIS-RELATED GENE 5) [Arabidopsis thalian	NP_177641	83	2	4.8	25236	0 (0)	0 (0)	0 (0)	1.035 (0.062)	0 (0)	0 (0)	2.564 (0.354)	1.71 (0.782)
120	22K antifungal protein - maize	JS0646	66	1	8.2	22028	0 (0)	0 (0)	0 (0)	1.006 (0.104)	0 (0)	0 (0)	0.738 (0.107)	1.308 (0.656)
126	PR5	NP_177641	83	2	4.8	25236	0 (0)	0 (0)	0.361 (0.016)	0 (0)	0 (0)	0 (0)	0 (0)	0 (0)
**Peroxidases**														
9	peroxidase	NP_201217	304	6	8.6	34868	1.642 (0.038)	0.12 (0.026)	0 (0)	1.922 (0.164)	0.631 (0.021)	0.407 (0.031)	0.33 (0.115)	0 (0)
17	peroxidase	NP_201217	104	2	8.6	34868	0.01 (0)	0 (0)	0.058 (0.004)	0 (0)	0.09 (0.017)	0.06 (0.036)	0 (0)	0 (0)
19	peroxidase	AAA32842	168	3	6	38148	0.001 (0.001)	0 (0)	0 (0)	0.069 (0.002)	0 (0)	0 (0)	0 (0)	0 (0)
20	peroxidase	CAA67092	68	2	8	34959	0.011 (0.001)	0.133 (0.04)	0 (0)	0.128 (0.01)	0 (0)	0 (0)	0 (0)	0 (0)
21	peroxidase	AAA32842	164	3	6	38148	0.156 (0.013)	0.027 (0.046)	0 (0)	0.125 (0.014)	0 (0)	0 (0)	0 (0)	0 (0)
32	peroxidase	AAA32842	163	3	6	38148	0.007 (0.001)	0.182 (0.029)	0 (0)	0 (0)	0 (0)	0 (0)	0 (0)	0 (0)
36	peroxidase	AAA32842	208	4	6	38148	2.222 (0.127)	1.743 (0.348)	0.88 (0.064)	0.488 (0.03)	0.674 (0.067)	0.777 (0.074)	0.184 (0.106)	0.065 (0.021)
37	peroxidase	CAA67092	241	5	8	34959	0.754 (0.038)	0.389 (0.051)	0 (0)	0.21 (0.036)	0.227 (0.047)	0.202 (0.171)	0 (0)	0 (0)
41	peroxidase	CAA67092	130	3	8	34959	0.776 (0.058)	0.514 (0.102)	0.136 (0.004)	0.049 (0.004)	0.289 (0.042)	0.357 (0.021)	0.035 (0.011)	0.025 (0.006)
44	peroxidase	CAA67335	104	2	6.9	36033	0.515 (0.015)	0.672 (0.042)	0.181 (0.016)	0.094 (0.005)	0.309 (0.31)	0.142 (0.035)	0 (0)	0 (0)
47	peroxidase	CAA67092	130	3	8	34959	0.414 (0.023)	0.228 (0.045)	0.16 (0.017)	0 (0)	0 (0)	0 (0)	0.629 (0.13)	0 (0)
48	Peroxidase C1C precursor	P15233	167	4	6.2	36525	0.168 (0.041)	0.273 (0.044)	0.244 (0.011)	0.117 (0.026)	0.178 (0.074)	0.097 (0.086)	0 (0)	0 (0)
49	peroxidase	NP_195469	451	8	8.6	35966	0.623 (0.026)	0 (0)	0.267 (0.01)	0 (0)	0 (0)	0.31 (0.115)	0 (0)	0 (0)
51	peroxidase	NP_195469	451	8	8.6	35966	0.506 (0.015)	0.208 (0.01)	0.413 (0.024)	0.1 (0.035)	0 (0)	0.987 (0.019)	0 (0)	0 (0)
53	peroxidase	NP_567919	158	5	8.6	35952	0.73 (0.02)	0.948 (0.123)	0.148 (0.007)	0.018 (0.004)	0 (0)	0.025 (0.004)	0 (0)	0 (0)
55	Peroxidase	CAA50677	254	4	8.1	38927	0.103 (0.007)	0.028 (0.024)	0.011 (0.004)	0 (0)	0.136 (0.064)	0.003 (0.003)	0 (0)	0 (0)
56	peroxidase	CAA67092	97	3	8	34959	2.996 (0.258)	1.827 (0.352)	2.278 (0.357)	0.006 (0.007)	0.582 (0.07)	0.611 (0.064)	0 (0)	0.053 (0.008)
60	peroxidase	CAA67092	238	5	8	34959	0.371 (0.016)	0.228 (0.041)	0 (0)	0 (0)	0 (0)	0.098 (0.003)	0.325 (0.088)	0 (0)
69	peroxidase	CAA07353	152	2	6.7	15966	1.039 (0.061)	0.997 (0.146)	1 (0.151)	1.312 (0.089)	4.047 (1.088)	1.716 (0.211)	6 (0.821)	1.168 (0.306)
72	peroxidase	NP_201217	361	7	8.6	34868	2.358 (0.183)	0.231 (0.033)	0.059 (0.006)	1.312 (0.153)	1.812 (0.418)	0.126 (0.032)	0.454 (0.066)	0.912 (0.217)
96	peroxidase	NP_201217	167	3	8.6	34868	0.041 (0.013)	0 (0)	0.204 (0.011)	0 (0)	0.218 (0.024)	0 (0)	0 (0)	0 (0)
100	peroxidase	CAA07353	146	3	6.7	15966	0.011 (0.003)	0 (0)	0 (0)	0 (0)	1.313 (0.517)	0 (0)	0 (0)	4.2 (0.455)
102	peroxidase	CAA07353	191	4	6.7	15966	0.084 (0.005)	0 (0)	0.016 (0)	0 (0)	0.03 (0.026)	0.009 (0.01)	0 (0)	0 (0)
103	peroxidase	CAA07353	147	3	6.7	15966	0.089 (0)	0.907 (0.085)	0.493 (0.019)	0.611 (0.028)	1.254 (0.236)	0.356 (0.229)	0 (0)	0 (0)
106	peroxidase	AAA32842	123	2	6	38148	0.054 (0.004)	0.015 (0.002)	0.064 (0.005)	0 (0)	0 (0)	0 (0)	0.408 (0.056)	0.142 (0.026)
110	peroxidase	NP_201217	87	2	8.6	34868	0.071 (0.007)	0.138 (0.01)	0.597 (0.028)	0.092 (0.005)	2.488 (0.22)	0.109 (0.054)	0.056 (0.007)	0 (0)
112	peroxidase	NP_201217	169	3	8.6	34868	2.246 (0.343)	3.068 (0.118)	3.08 (0.192)	1.519 (0.095)	2.653 (0.336)	1.942 (0.35)	0.594 (0.023)	0.26 (0.035)
115	peroxidase.	AAA32842	156	3	6	38148	0 (0)	0 (0)	0 (0)	0 (0)	0 (0)	0 (0)	0.43 (0.071)	0.66 (0.076)
116	peroxidase	CAA67092	238	5	8	34959	0 (0)	0 (0)	0 (0)	0 (0)	0 (0)	0 (0)	0.806 (0.026)	0 (0)
**Hydrolases/Transferases**														
3	23S rRNA m(2)G2445 methyltransferase	YP_113222	64	2	9.2	82978	0.896 (0.247)	0.005 (0.004)	0.806 (0.036)	0.416 (0.008)	0 (0)	0 (0)	0.007 (0.002)	0.004 (0.006)
11	pectin methylesterase-like protein	BAB09534	60	2	6.6	63463	0.091 (0.017)	0.189 (0.027)	0.392 (0.01)	0.018 (0.004)	0.005 (0.003)	0.073 (0.018)	0 (0)	0 (0)
13	hydrolase	NP_194843	80	2	5.8	52682	0.085 (0.007)	0.032 (0.028)	0.025 (0.004)	0.07 (0.022)	0.006 (0.007)	0.193 (0.012)	0.041 (0.004)	0 (0)
14	Glycosyl hydrolase family 14 (beta-amylase)	NP_567460	230	5	5.2	56027	0.015 (0.001)	0 (0)	0.009 (0.015)	0 (0)	0.024 (0.004)	0.193 (0.084)	0 (0)	0 (0)
16	glycosyl hydrolase family 18 protein	NP_193717	63	2	9.4	40848	0.09 (0.026)	0 (0)	0 (0)	0 (0)	0 (0)	1.23 (0.364)	0.032 (0.008)	0.036 (0.009)
29	Lipase/Acylhydrolase with GDSL-motif	AAD25774	278	5	6.5	43848	0.653 (0.008)	0.518 (0.031)	0.07 (0.012)	0.288 (0.021)	0 (0)	0 (0)	0.454 (0.056)	0.057 (0.011)
35	Lipase/Acylhydrolase with GDSL-motif	AAD25773	561	11	6.1	47699	0.675 (0.065)	0.389 (0.027)	0.097 (0.012)	0.046 (0.007)	0.271 (0.008)	0.288 (0.051)	0.117 (0.032)	1.902 (0.045)
39	Belongs to the PF|00657 Lipase/Acylhydrolase with GDSL-motif f	AAD25773	332	7	6.1	47699	0.212 (0.042)	0.029 (0.025)	0 (0)	0 (0)	0.034 (0.007)	0.082 (0.016)	0.138 (0.092)	0 (0)
40	hydrolase	NP_194413	78	2	8.1	48523	0.424 (0.014)	0.211 (0.023)	1.532 (0.098)	0.492 (0.018)	0.145 (0.009)	0.974 (0.056)	0.51 (0.087)	0.488 (0.026)
82	Glycosyl hydrolase family 17 (endo-1,3 β-glucanase)	NP_193361	155	4	5.3	33565	0.201 (0.015)	0.294 (0.037)	0.066 (0.007)	0.005 (0.005)	0 (0)	0.047 (0.009)	0.113 (0.082)	1.101 (0.146)
**Miscellaneous function-related**														
6	Predicted metalloendopeptidase	ZP_0037864	68	2	4.6	72082	0.186 (0.018)	0.273 (0.037)	0.144 (0.002)	0 (0)	0.337 (0.01)	0 (0)	0.125 (0.047)	0.021 (0.012)
7	XYL4	NP_201262	295	5	7.8	84255	0.72 (0.034)	0.391 (0.027)	0.276 (0.008)	0.541 (0.02)	0.092 (0.007)	0.154 (0.048)	0.087 (0.029)	0.003 (0.005)
8	XYL4	NP_201262	295	5	7.8	84255	0.197 (0.018)	0.022 (0.02)	0.029 (0.003)	0.061 (0.004)	0.002 (0.003)	0 (0)	0 (0)	0 (0)
10	SKS5 (SKU5 Similar 5	NP_177743	365	8	8.5	60003	0.696 (0.073)	0.53 (0.065)	0.806 (0.013)	0.734 (0.058)	0.272 (0.029)	0.174 (0.103)	0.062 (0.032)	0.043 (0.019)
12	Eukaryotic aspartyl protease	AAF29389	94	2	6.1	42367	0.219 (0.026)	0.025 (0.021)	0.122 (0.022)	0.084 (0.025)	0.016 (0.004)	0.053 (0.008)	0.003 (0.005)	0.009 (0.003)
15	pepsin A	NP_563851	251	7	7.5	47613	3.06 (0.121)	0.79 (0.113)	0.824 (0.019)	1.268 (0.239)	0.276 (0.062)	0.057 (0.01)	0 (0)	0 (0)
25	protein kinase	NP_194048	93	2	6.6	29703	0.595 (0.078)	0.286 (0.036)	0.057 (0.002)	0.069 (0.011)	0 (0)	0.013 (0.022)	0.155 (0.054)	0 (0)
26	CORI3	NP_194091	87	2	5.9	47009	0.203 (0.072)	0.091 (0.034)	0.035 (0.003)	0.08 (0.017)	0 (0)	0 (0)	0 (0)	0 (0)
28	CORI3	NP_194091	401	9	5.9	47009	1.351 (0.205)	0.743 (0.132)	0.427 (0.024)	0.223 (0.044)	0.207 (0.024)	0.208 (0.07)	0.155 (0.039)	0.031 (0.003)
34	pepsin A	NP_188478	278	5	5.3	53201	2.325 (0.33)	0.238 (0.027)	1.992 (0.129)	0.54 (0.052)	0.178 (0.009)	1.236 (0.256)	0.271 (0.063)	0.112 (0.026)
67	MERI5B (MERISTEM-5);	NP_194756	374	7	8.4	30736	2.47 (0.137)	2.785 (0.233)	1.299 (0.271)	2.822 (0.154)	1.683 (0.151)	0.913 (0.078)	1.343 (0.189)	3.612 (0.46)
68	F12M16.14	AAF69549	84	2	8.5	36968	0.034 (0.007)	1.359 (0.225)	0.027 (0.007)	0 (0)	0.134 (0.013)	0.122 (0.029)	0.12 (0.051)	0.014 (0.001)
70	XTR6	NP_194311	409	8	5.1	32043	0.267 (0.034)	0.689 (0.055)	0 (0)	0.393 (0.032)	0.074 (0.029)	0 (0)	1.679 (0.605)	0 (0)

71	putative mitochondrial NAD-dependent malate dehydrogenase	CAD33240	66	2	8.9	36172	0.002 (0.003)	0.189 (0.019)	0.004 (0.004)	0.014 (0.007)	0 (0)	0.048 (0.003)	0 (0)	0.351 (0.055)
73	XYL 6	NP_194311	228	4	5.1	32043	0.077 (0.008)	0.689 (0.059)	0.809 (0.022)	0.51 (0.088)	0 (0)	0.843 (0.119)	0.013 (0.007)	1.124 (0.159)
76	ATGLX1	NP_172648	255	5	5.2	31908	0.208 (0.01)	0 (0)	0 (0)	0 (0)	0 (0)	0 (0)	0 (0)	0.193 (0.022)
77	MERI5B; hydrolase, acting on glycosyl bonds	NP_194756	393	8	8.4	30736	0.04 (0.017)	0.401 (0.037)	0 (0)	0.797 (0.2)	0.212 (0.051)	0.287 (0.039)	1.233 (0.269)	3.621 (0.576)
78	MERI5B; hydrolase, acting on glycosyl bonds	NP_194756	377	9	8.4	30736	2.16 (0.075)	1.825 (0.151)	1.548 (0.081)	1.1 (0.146)	1.412 (0.427)	0.874 (0.109)	3.142 (0.845)	0 (0)
79	Meri-5	CAA58001	76	2	9	19482	0.22 (0.045)	0 (0)	0 (0)	0.311 (0.06)	0 (0)	0.182 (0.041)	2.136 (0.457)	0 (0)
85	ATEXLA1 (ARABIDOPSIS THALIANA EXPANSIN-LIKE A1)	NP_190183	71	2	8.3	28683	0.209 (0.021)	1.01 (0.01)	1.366 (0.072)	1.1 (0.207)	0.223 (0.04)	0.987 (0.358)	1.354 (0.338)	1.882 (0.258)
95	SJCHGC08196 protein	AAX26197	72	2	9	22913	0.003 (0.005)	0 (0)	0 (0)	0 (0)	0 (0)	0 (0)	0 (0)	0 (0)
111	LOC683313 protein [Rattus norvegicus].	AAH99121	247	4	8.1	59213	0.331 (0.057)	0 (0)	0.044 (0.004)	0 (0)	0.597 (0.047)	0.328 (0.113)	0 (0)	0 (0)
117	CG4810	NP_731675	63	1	6	62709	0 (0)	0 (0)	0 (0)	0 (0)	0 (0)	0 (0)	0.109 (0.018)	0.11 (0.04)
128	XYL4 (beta-xylosidase 4);	NP_201262	89	2	7.8	84255	0.11 (0.045)	0 (0)	0.024 (0.005)	0.141 (0.027)	0.037 (0.028)	0.081 (0.026)	0 (0)	0 (0)
**Secretory protein-related**														
74	33 kDa secretory protein-related	NP_199665	106	2	8.9	28977	0.005 (0.004)	0 (0)	0 (0)	0.444 (0.051)	0.004 (0.007)	0 (0)	0 (0)	0.22 (0.052)
94	secretory protein	NP_565369	105	3	8.6	25149	0.006 (0.006)	0.063 (0.009)	0.114 (0.032)	0 (0)	0.02 (0.034)	0 (0)	0.149 (0.018)	0 (0)
**Unknown function**														
18	putative protein	CAA18827	260	6	5.2	35417	0.245 (0.016)	0.122 (0.027)	0.154 (0.014)	0.504 (0.024)	0 (0)	0.428 (0.046)	0.002 (0.003)	0 (0)
22	hypothetical protein MG05843.4	XP_369621	67	2	9.3	51690	0.132 (0.029)	0 (0)	0.491 (0.053)	0.285 (0.014)	0 (0)	1.23 (0.329)	0.378 (0.04)	0.585 (0.123)
23	putative protein	CAA18827	212	5	5.2	35417	0.411 (0.011)	0 (0)	0.491 (0.025)	0.169 (0.018)	0.022 (0.005)	1.175 (0.274)	0.336 (0.052)	0.008 (0.005)
24	putative protein	CAA18827	340	7	5.2	35417	1.456 (0.126)	1.123 (0.179)	1.3 (0.364)	0.442 (0.038)	1.128 (0.133)	0.844 (0.154)	0.5 (0.146)	0.322 (0.075)
33	unknown protein	AAL38777	204	6	8.7	50323	0.964 (0.029)	0.718 (0.071)	0.28 (0.014)	0 (0)	0 (0)	0 (0)	0.628 (0.155)	0 (0)
43	unknown protein (Jacalin-like lectin domain)	NP_175618	391	8	5.4	31776	0.532 (0.059)	0 (0)	0 (0)	0 (0)	0 (0)	0.022 (0.019)	0.095 (0.016)	0.263 (0.043)
50	Unknown protein	NP_175618	349	7	5.4	31776	2.988 (0.188)	2.33 (0.265)	0.272 (0.009)	0 (0)	0 (0)	0 (0)	0.064 (0.015)	0 (0)
52	unknown protein	AAL38777	95	2	8.7	50323	0.456 (0.017)	0.919 (0.103)	0 (0)	0.061 (0.007)	1.367 (0.884)	1.886 (0.909)	0.368 (0.041)	0.24 (0.058)
59	unknown protein	NP_175618	518	10	5.4	31776	2.456 (0.301)	1.692 (0.347)	1.436 (0.155)	2.391 (0.245)	2.085 (0.298)	3.884 (1.26)	1.842 (0.195)	0.028 (0.006)
61	unknown protein	NP_565779	349	5	8.8	43326	0.223 (0.018)	0 (0)	0.044 (0.004)	0.14 (0.045)	0.318 (0.023)	0.121 (0.025)	0.233 (0.058)	0 (0)
63	unknown protein	NP_175618	136	3	5.4	31776	0.022 (0.003)	1.074 (0.141)	1.058 (0.056)	0.903 (0.026)	0.15 (0.104)	1.241 (0.277)	0.176 (0.025)	0.811 (0.087)
81	unknown protein	NP_175618	175	4	5.4	31776	0.534 (0.03)	1.01 (0.147)	1 (0.12)	1.1 (0.194)	2.738 (0.265)	0 (0)	0.658 (0.076)	1.882 (0.378)
87	Unknown protein	AAM65935	72	2	5.5	32138	0.076 (0.007)	0.217 (0.02)	0.058 (0.007)	0.03 (0.022)	0.062 (0.036)	0.156 (0.047)	0.082 (0.036)	0 (0)
89	unknown protein	NP_565369	400	7	8.6	25149	1.098 (0.02)	0.922 (0.06)	0.913 (0.013)	0.045 (0.006)	0.939 (0.404)	2.203 (0.372)	0 (0)	0.446 (0.159)
90	unknown protein	NP_565369	118	3	8.6	25149	0.007 (0.002)	0.046 (0.007)	0.092 (0.005)	0 (0)	0.005 (0.004)	0.095 (0.054)	0.31 (0.057)	1.308 (0.524)
93	Putative Lectin	AAM65935	98	3	5.5	32138	3.048 (0.346)	5.502 (0.405)	1.144 (0.146)	0.634 (0.033)	0 (0)	0.399 (0.063)	0.078 (0.021)	0.142 (0.073)
109	unknown protein	AAL38777	204	6	8.7	50323	0.122 (0.035)	0.091 (0.003)	0.389 (0.009)	0 (0)	0 (0)	0.052 (0.007)	0.058 (0.014)	0 (0)
114	unnamed protein product [Homo sapiens].	CAA32649	595	11	5.2	59492	0.118 (0.028)	0.129 (0.011)	0.004 (0.004)	0 (0)	1.707 (0.263)	0.171 (0.025)	0 (0)	0 (0)
119	unknown protein	NP_175618	61	1	5.4	31776	3.971 (0.192)	7.868 (0.309)	5.367 (0.196)	1.225 (0.476)	2.223 (0.579)	5.323 (1.203)	1.796 (0.217)	1.158 (0.259)
121	hypothetical protein Sfum_4078	YP_848178	64	2	4.8	22084	0.099 (0.02)	1.184 (0.027)	0.951 (0.037)	0.497 (0.095)	0 (0)	0 (0)	1.56 (0.268)	2.667 (0.444)
122	unknown protein	NP_179117	171	4	6	25345	0 (0)	0 (0)	0 (0)	1.12 (0.158)	0 (0)	0 (0)	2.33 (0.262)	1.17 (0.729)
123	putative protein	CAA18827	100	3	5.2	35417	0 (0)	0 (0)	0 (0)	0.237 (0.025)	0 (0)	0 (0)	0 (0)	0 (0)
124	unknown protein	NP_175618	183	3	5.4	31776	0.533 (0.061)	1.981 (0.124)	0.28 (0.045)	1.545 (0.091)	0 (0)	1.886 (0.294)	0 (0)	0 (0)
