# Supplementary material for: Root Secreted Metabolites and Proteins Are Involved in the Early Events of Plant-Plant Recognition Prior to Competition
Source: PLoS One. 2012 Oct 2;7(10):e46640. doi: 10.1371/journal.pone.0046640 (PMC3462798; doi:10.1371/journal.pone.0046640)
Supplement: Table S3 — Univariate ANOVA comparing each individual protein across treatments. (PDF) [file pone.0046640.s005.pdf]

Table S3. Univariate ANOVA comparing each individual protein across treatments.

	Univariate ANOVA							
	Model			Residual				
**Spot #**	**SS**	**df**	**MS**	**SS**	**df**	**MS**	**F-value**	**Pr> F**
**Myrosinases**								
1	3.04E+00	7	4.34E-01	3.71E-02	16	0.0023	187.1	<0.0001
2	2.58E+00	7	3.68E-01	5.95E-02	16	0.0037	99.0	<0.0001
27	2.79E-02	7	3.98E-03	2.56E-04	16	2E-05	249.0	<0.0001
31	3.86E-01	7	5.52E-02	3.43E-03	16	0.0002	257.4	<0.0001
42	3.48E+00	7	4.97E-01	9.20E-02	16	0.0058	86.4	<0.0001
58	3.93E-01	7	5.62E-02	1.08E-01	16	0.0067	8.3	0.0002
92	8.92E-01	7	1.27E-01	1.40E-02	16	0.0009	146.0	<0.0001
97	1.13E-01	7	1.62E-02	1.62E-03	16	0.0001	159.3	<0.0001
98	5.10E-01	7	7.29E-02	8.52E-03	16	0.0005	136.8	<0.0001
99	2.99E+00	7	4.27E-01	1.18E-01	16	0.0074	57.7	<0.0001
101	1.86E+00	7	2.66E-01	1.86E-01	16	0.0116	22.9	<0.0001
104	1.52E+01	7	2.18E+00	1.31E+00	16	0.0821	26.5	<0.0001
105	2.88E+00	7	4.12E-01	6.65E-03	16	0.0004	991.6	<0.0001
107	7.36E-01	7	1.05E-01	1.14E-02	16	0.0007	147.3	<0.0001
108	3.85E-01	7	5.49E-02	3.35E-03	16	0.0002	262.5	<0.0001
125	1.41E+00	7	2.01E-01	2.65E-02	16	0.0017	121.0	<0.0001
127	8.08E-01	7	1.15E-01	6.05E-03	16	0.0004	305.4	<0.0001
**Defense-related proteins**								
4	8.17E-02	7	1.17E-02	4.80E-03	16	0.0003	38.9	<0.0001
5	4.68E-01	7	6.68E-02	3.22E-02	16	0.002	33.2	<0.0001
30	1.46E-01	7	2.08E-02	7.50E-03	16	0.0005	44.5	<0.0001
38	1.79E-01	7	2.56E-02	5.83E-03	16	0.0004	70.3	<0.0001
45	3.99E+00	7	5.70E-01	1.08E+00	16	0.0676	8.4	0.0002
46	1.87E-02	7	2.68E-03	6.68E-04	16	4E-05	64.1	<0.0001
54	1.41E-02	7	2.02E-03	6.88E-04	16	4E-05	47.0	<0.0001
57	3.11E-01	7	4.44E-02	1.43E-03	16	9E-05	498.2	<0.0001
62	4.72E+00	7	6.74E-01	2.56E-01	16	0.016	42.2	<0.0001
64	6.00E+00	7	8.57E-01	6.49E-01	16	0.0406	21.1	<0.0001
65	4.73E+00	7	6.76E-01	2.97E-01	16	0.0185	36.5	<0.0001
66	7.61E+01	7	1.09E+01	1.51E+00	16	0.0943	115.3	<0.0001
75	2.11E+01	7	3.02E+00	2.52E+00	16	0.1574	19.2	<0.0001
80	1.10E+01	7	1.56E+00	2.76E-01	16	0.0173	90.7	<0.0001
83	5.55E-02	7	7.93E-03	7.57E-03	16	0.0005	16.8	<0.0001
84	1.51E+00	7	2.15E-01	1.11E-02	16	0.0007	311.3	<0.0001
86	5.54E+00	7	7.91E-01	6.65E-02	16	0.0042	190.5	<0.0001
88	4.52E+01	7	6.46E+00	1.92E+00	16	0.1199	53.9	<0.0001

91	1.51E+00	7	2.16E-01	1.56E-01	16	0.0098	22.1	<0.0001
113	1.13E+02	7	1.62E+01	3.25E+00	16	0.2032	79.7	<0.0001
118	2.11E+01	7	3.02E+00	1.48E+00	16	0.0928	32.6	<0.0001
120	6.31E+00	7	9.01E-01	9.06E-01	16	0.0566	15.9	<0.0001
126	3.42E-01	7	4.89E-02	5.54E-04	16	3E-05	1411.4	<0.0001
**Peroxidases**								
9	1.17E+01	7	1.67E+00	8.80E-02	16	0.0055	303.0	<0.0001
17	2.77E-02	7	3.95E-03	3.24E-03	16	0.0002	19.5	<0.0001
19	1.24E-02	7	1.78E-03	1.60E-05	16	1E-06	1778.4	<0.0001
20	7.48E-02	7	1.07E-02	3.45E-03	16	0.0002	49.5	<0.0001
21	8.65E-02	7	1.24E-02	5.17E-03	16	0.0003	38.2	<0.0001
32	8.61E-02	7	1.23E-02	1.69E-03	16	0.0001	116.5	<0.0001
36	1.17E+01	7	1.67E+00	3.29E-01	16	0.0206	81.4	<0.0001
37	1.38E+00	7	1.97E-01	7.40E-02	16	0.0046	42.5	<0.0001
41	1.52E+00	7	2.17E-01	3.29E-02	16	0.0021	105.5	<0.0001
44	1.25E+00	7	1.79E-01	2.01E-01	16	0.0125	14.2	<0.0001
47	1.17E+00	7	1.67E-01	3.99E-02	16	0.0025	66.7	<0.0001
48	2.16E-01	7	3.09E-02	3.52E-02	16	0.0022	14.0	<0.0001
49	1.13E+00	7	1.61E-01	2.82E-02	16	0.0018	91.4	<0.0001
51	2.52E+00	7	3.61E-01	5.21E-03	16	0.0003	1106.4	<0.0001
53	3.05E+00	7	4.36E-01	3.16E-02	16	0.002	220.9	<0.0001
55	6.04E-02	7	8.63E-03	9.63E-03	16	0.0006	14.3	<0.0001
56	2.85E+01	7	4.07E+00	6.55E-01	16	0.041	99.4	<0.0001
60	5.23E-01	7	7.47E-02	1.96E-02	16	0.0012	61.0	<0.0001
69	7.25E+01	7	1.04E+01	4.11E+00	16	0.2568	40.3	<0.0001
72	1.52E+01	7	2.18E+00	5.72E-01	16	0.0358	60.9	<0.0001
96	1.92E-01	7	2.74E-02	1.82E-03	16	0.0001	240.7	<0.0001
100	4.66E+01	7	6.66E+00	9.50E-01	16	0.0594	112.3	<0.0001
102	1.76E-02	7	2.52E-03	1.70E-03	16	0.0001	23.8	<0.0001
103	4.28E+00	7	6.11E-01	2.34E-01	16	0.0146	41.7	<0.0001
106	4.07E-01	7	5.81E-02	7.80E-03	16	0.0005	119.1	<0.0001
110	1.51E+01	7	2.15E+00	1.05E-01	16	0.0066	326.5	<0.0001
112	2.39E+01	7	3.42E+00	8.32E-01	16	0.052	65.8	<0.0001
115	1.42E+00	7	2.02E-01	2.18E-02	16	0.0014	148.1	<0.0001
116	1.71E+00	7	2.44E-01	1.37E-03	16	9E-05	2849.3	<0.0001
**Hydrolases/Transferases**								
3	3.17E+00	7	4.53E-01	1.26E-01	16	0.0079	57.6	<0.0001
11	3.89E-01	7	5.56E-02	3.11E-03	16	0.0002	286.2	<0.0001
13	8.16E-02	7	1.17E-02	3.31E-03	16	0.0002	56.4	<0.0001
14	9.26E-02	7	1.32E-02	1.47E-02	16	0.0009	14.4	<0.0001
16	3.85E+00	7	5.50E-01	2.68E-01	16	0.0167	32.9	<0.0001
29	1.42E+00	7	2.02E-01	9.99E-03	16	0.0006	323.9	<0.0001

35	7.85E+00	7	1.12E+00	2.24E-02	16	0.0014	801.6	<0.0001
39	1.26E-01	7	1.80E-02	2.27E-02	16	0.0014	12.7	<0.0001
40	4.29E+00	7	6.13E-01	4.49E-02	16	0.0028	218.2	<0.0001
82	2.82E+00	7	4.03E-01	6.01E-02	16	0.0038	107.4	<0.0001
**Miscellaneous function-related**								
6	3.36E-01	7	4.80E-02	8.63E-03	16	0.0005	89.0	<0.0001
7	1.32E+00	7	1.88E-01	1.15E-02	16	0.0007	261.8	<0.0001
8	9.53E-02	7	1.36E-02	1.59E-03	16	1E-04	137.4	<0.0001
10	2.07E+00	7	2.95E-01	5.27E-02	16	0.0033	89.5	<0.0001
12	1.15E-01	7	1.65E-02	4.88E-03	16	0.0003	54.0	<0.0001
15	2.23E+01	7	3.18E+00	1.78E-01	16	0.0112	285.6	<0.0001
25	8.86E-01	7	1.27E-01	2.23E-02	16	0.0014	91.0	<0.0001
26	1.09E-01	7	1.55E-02	1.34E-02	16	0.0008	18.6	<0.0001
28	3.97E+00	7	5.66E-01	1.39E-01	16	0.0087	65.3	<0.0001
34	1.63E+01	7	2.33E+00	4.00E-01	16	0.025	93.0	<0.0001
67	1.86E+01	7	2.66E+00	8.97E-01	16	0.0561	47.5	<0.0001
68	4.46E+00	7	6.37E-01	1.09E-01	16	0.0068	93.2	<0.0001
70	6.97E+00	7	9.95E-01	7.47E-01	16	0.0467	21.3	<0.0001
71	3.46E-01	7	4.94E-02	7.15E-03	16	0.0004	110.5	<0.0001
73	3.91E+00	7	5.59E-01	1.03E-01	16	0.0065	86.5	<0.0001
76	1.81E-01	7	2.59E-02	1.25E-03	16	8E-05	332.5	<0.0001
77	3.04E+01	7	4.34E+00	9.02E-01	16	0.0564	77.0	<0.0001
78	1.81E+01	7	2.59E+00	1.93E+00	16	0.1209	21.4	<0.0001
79	1.12E+01	7	1.60E+00	4.33E-01	16	0.0271	59.0	<0.0001
85	6.82E+00	7	9.75E-01	7.20E-01	16	0.045	21.7	<0.0001
95	2.36E-05	7	3.38E-06	5.40E-05	16	3E-06	1.0	0.4663
111	1.09E+00	7	1.56E-01	3.68E-02	16	0.0023	67.9	<0.0001
117	5.40E-02	7	7.71E-03	3.92E-03	16	0.0002	31.4	<0.0001
128	6.35E-02	7	9.08E-03	8.82E-03	16	0.0006	16.5	<0.0001
**Secretory protein-related**								
74	5.67E-01	7	8.10E-02	1.09E-02	16	0.0007	119.0	<0.0001
94	7.23E-02	7	1.03E-02	5.42E-03	16	0.0003	30.5	<0.0001
**Unknown function**								
18	8.14E-01	7	1.16E-01	7.96E-03	16	0.0005	233.8	<0.0001
22	3.41E+00	7	4.87E-01	2.59E-01	16	0.0162	30.1	<0.0001
23	3.24E+00	7	4.63E-01	1.59E-01	16	0.0099	46.7	<0.0001
24	3.83E+00	7	5.48E-01	5.03E-01	16	0.0315	17.4	<0.0001
33	3.24E+00	7	4.62E-01	6.07E-02	16	0.0038	122.0	<0.0001
43	7.73E-01	7	1.10E-01	1.22E-02	16	0.0008	144.6	<0.0001
50	3.13E+01	7	4.47E+00	2.13E-01	16	0.0133	336.8	<0.0001
52	9.50E+00	7	1.36E+00	3.25E+00	16	0.2031	6.7	0.0008
59	2.47E+01	7	3.53E+00	4.02E+00	16	0.2515	14.0	<0.0001

61	2.87E-01	7	4.11E-02	1.42E-02	16	0.0009	46.3	<0.0001
63	4.94E+00	7	7.06E-01	2.40E-01	16	0.015	47.1	<0.0001
81	1.51E+01	7	2.16E+00	5.88E-01	16	0.0367	58.7	<0.0001
87	1.02E-01	7	1.46E-02	1.17E-02	16	0.0007	19.8	<0.0001
89	1.03E+01	7	1.47E+00	6.64E-01	16	0.0415	35.5	<0.0001
90	4.18E+00	7	5.97E-01	5.63E-01	16	0.0352	17.0	<0.0001
93	7.94E+01	7	1.13E+01	6.34E-01	16	0.0396	286.3	<0.0001
109	3.52E-01	7	5.02E-02	3.23E-03	16	0.0002	248.9	<0.0001
114	7.22E+00	7	1.03E+00	1.42E-01	16	0.0089	116.4	<0.0001
119	1.24E+02	7	1.77E+01	4.60E+00	16	0.2873	61.5	<0.0001
121	1.82E+01	7	2.60E+00	5.63E-01	16	0.0352	73.8	<0.0001
122	1.62E+01	7	2.31E+00	1.25E+00	16	0.0783	29.5	<0.0001
123	1.47E-01	7	2.11E-02	1.34E-03	16	8E-05	251.9	<0.0001
124	1.62E+01	7	2.31E+00	2.32E-01	16	0.0145	159.0	<0.0001
