# Supplementary material for: Root Secreted Metabolites and Proteins Are Involved in the Early Events of Plant-Plant Recognition Prior to Competition
Source: PLoS One. 2012 Oct 2;7(10):e46640. doi: 10.1371/journal.pone.0046640 (PMC3462798; doi:10.1371/journal.pone.0046640)
Supplement: Table S4 — Total secreted proteins by category of individually grown or plants co-cultured with homologous or different individuals. (PDF) [file pone.0046640.s006.pdf]

Table S4. Total secreted proteins by category in individually grown or plants co-cultured with homologous or different individuals. All reported values are the arithmetic mean (n = 3) and standard deviation.

	Protein Spot Intensity							
**Category**	**Col**	**Col-Col**	**Col-Ler**	**Col-Cap**	**Ler**	**Ler-Ler**	**Cap**	**Cap-Cap**
Myrosinases	6.192 (0.172)	4.954 (0.241)	5.985 (0.158)	3.583 (0.1)	5.424 (0.756)	4.725 (0.587)	3.297 (0.539)	3.345 (0.679)
Defense-related proteins	8.767 (0.079)	13.388 (0.721)	15.867 (0.279)	17.204 (0.665)	20.496 (1.01)	9.826 (1.024)	14.106 (1.64)	12.557 (0.761)
Peroxidases	17.998 (0.46)	12.876 (0.491)	10.289 (0.128)	8.172 (0.382)	16.931 (1.57)	8.334 (0.579)	10.251 (1.072)	7.485 (1.018)
Hydrolases/Transferases	3.342 (0.265)	1.667 (0.054)	2.997 (0.139)	1.335 (0.043)	0.485 (0.019)	3.08 (0.436)	1.412 (0.195)	3.588 (0.128)
Miscellaneous function-related	15.683 (0.553)	12.336 (0.32)	9.833 (0.139)	10.788 (0.549)	5.752 (0.633)	6.56 (0.187)	11.987 (2.197)	11.126 (0.264)
Secretory protein-related	0.011 (0.009)	0.063 (0.01)	0.114 (0.032)	0.444 (0.051)	0.024 (0.031)	0 (0)	0.149 (0.018)	0.22 (0.052)
Unknown function	19.491 (0.566)	26.928 (0.239)	15.724 (0.772)	11.329 (0.703)	12.744 (1.137)	21.116 (2.707)	11.494 (0.084)	11.03 (1.199)
