# Supplementary material for: Root Secreted Metabolites and Proteins Are Involved in the Early Events of Plant-Plant Recognition Prior to Competition
Source: PLoS One. 2012 Oct 2;7(10):e46640. doi: 10.1371/journal.pone.0046640 (PMC3462798; doi:10.1371/journal.pone.0046640)
Supplement: Table S5 — Univariate ANOVA comparing each protein category across treatments. (PDF) [file pone.0046640.s007.pdf]

**Table S5. Univariate ANOVA comparing each protein category across treatments.**

| | Univariate ANOVA | | | | | | | |
|---|---|---|---|---|---|---|---|---|
| | Model | | | Residual | | | | |
| Category | SS | df | MS | SS | df | MS | F-value | Pr> F |
| Myrosinases | 28.553 | 7 | 4.079 | 3.585 | 16 | 0.224 | 18.20 | <0.0001 |
| Defense-related proteins | 309.668 | 7 | 44.238 | 12.774 | 16 | 0.798 | 55.41 | <0.0001 |
| Peroxidases | 341.566 | 7 | 48.795 | 11.213 | 16 | 0.701 | 69.63 | <0.0001 |
| Hydrolases/Transferases | 27.664 | 7 | 3.952 | 0.682 | 16 | 0.043 | 92.75 | <0.0001 |
| Miscellaneous function-related | 214.261 | 7 | 30.609 | 12.131 | 16 | 0.758 | 40.37 | <0.0001 |
| Secretory protein-related | 0.462 | 7 | 0.066 | 0.016 | 16 | 0.001 | 66.55 | <0.0001 |
| Unknown function | 704.612 | 7 | 100.659 | 23.081 | 16 | 1.443 | 69.78 | <0.0001 |
