# Supplementary material for: Root Secreted Metabolites and Proteins Are Involved in the Early Events of Plant-Plant Recognition Prior to Competition
Source: PLoS One. 2012 Oct 2;7(10):e46640. doi: 10.1371/journal.pone.0046640 (PMC3462798; doi:10.1371/journal.pone.0046640)
Supplement: Table S6 — Multivariate ANOVA comparing protein profiles by category across treatments. (PDF) [file pone.0046640.s008.pdf]

Table S6. Multivarite ANOVA comparing protein profiles by category across treatments.

Multivariate ANOVA (Wilks' Lambda)

Effect	Value	F-value	Num df	Den df	Pr> F
Treatment	0.0000	68.6	49	55.19	<0.0001
**Contrast**					
Col vs. Ler	0.0061	231.15	7	10	<0.0001
Col vs. Cap	0.0121	116.57	7	10	<0.0001
Col vs. Col-Col	0.0101	140.11	7	10	<0.0001
Col vs. Col-Ler	0.0108	130.95	7	10	<0.0001
Col vs. Col-Cap	0.0067	210.24	7	10	<0.0001
Col vs. Ler-Ler	0.0133	105.67	7	10	<0.0001
Col vs. Cap-Cap	0.0125	112.49	7	10	<0.0001
Ler vs. Cap	0.0232	60.09	7	10	<0.0001
Ler vs. Col-Col	0.0137	103.19	7	10	<0.0001
Ler vs. Col-Ler	0.0253	55.08	7	10	<0.0001
Ler vs. Col-Cap	0.0170	82.74	7	10	<0.0001
Ler vs. Ler-Ler	0.0152	92.80	7	10	<0.0001
Ler vs. Cap-Cap	0.0099	143.30	7	10	<0.0001
Cap vs. Col-Col	0.0302	45.88	7	10	<0.0001
Cap vs. Col-Ler	0.0755	17.48	7	10	<0.0001
Cap vs. Col-Cap	0.0639	20.92	7	10	<0.0001
Cap vs. Ler-Ler	0.0440	31.01	7	10	<0.0001
Cap vs. Cap-Cap	0.0363	37.89	7	10	<0.0001
Col-Col vs. Col-Ler	0.0212	65.98	7	10	<0.0001
Col-Col vs. Col-Cap	0.0175	80.04	7	10	<0.0001
Col-Col vs. Ler-Ler	0.0296	46.76	7	10	<0.0001
Col-Col vs. Cap-Cap	0.0110	128.05	7	10	<0.0001
Col-Cap vs. Col-Ler	0.0330	41.91	7	10	<0.0001
Col-Cap vs. Ler-Ler	0.0200	70.01	7	10	<0.0001
Col-Cap vs. Cap-Cap	0.0236	59.00	7	10	<0.0001
Col-Ler vs Ler-Ler	0.0870	14.99	7	10	0.0001
Col-Ler vs. Cap-Cap	0.0436	31.31	7	10	<0.0001
Ler-Ler vs Cap-Cap	0.0332	41.61	7	10	<0.0001
